# One-Year Outcomes After Traumatic Brain Injury and Early Extracranial Surgery in the TRACK-TBI Study

**DOI:** 10.1001/jamanetworkopen.2025.37271

**Published:** 2025-10-10

**Authors:** Christopher J. Roberts, Amelia W. Maiga, Jason Barber, Nancy R. Temkin, Ruturaj Vala, Mayur B. Patel, Claudia S. Robertson, Alex B. Valadka, John K. Yue, Amy J. Markowitz, Geoffrey T. Manley, Lindsay D. Nelson, and the Transforming Clinical Research and Knowledge in TBI (TRACK-TBI) Investigators, Ann-Christine Duhaime, Brandon Foreman, Ramesh Grandhi, C. Dirk Keene, Vijay Krishnamoorthy, Christine Mac Donald, Michael McCrea, Randall Merchant, Laura B. Ngwenya, Ava Puccio, Richard B. Rodgers, David Schnyer, Sabrina R. Taylor, Ross Zafonte

**Affiliations:** 1 Department of Anesthesiology, Medical College of Wisconsin, Milwaukee; 2 Department of Anesthesiology, Zablocki Veterans Affairs Medical Center, Milwaukee, Wisconsin; 3 Division of Acute Care Surgery, Department of Surgery, Section of Surgical Sciences, Vanderbilt University Medical Center, Nashville, Tennessee; 4 Critical Illness, Brain Dysfunction, and Survivorship Center, Center for Health Services Research, Vanderbilt University Medical Center, Nashville, Tennessee; 5 Surgical Services, Nashville VA Medical Center, Tennessee Valley Healthcare System, Nashville; 6 Department of Neurological Surgery, University of Washington, Seattle; 7 Department of Biostatistics, University of Washington, Seattle; 8 Department of Neurosurgery, Baylor College of Medicine, Houston, Texas; 9 Department of Neurological Surgery; University of Texas Southwestern Medical Center, Dallas; 10 Department of Neurological Surgery, University of California, San Francisco; 11 Brain and Spinal Injury Center, San Francisco, California; 12 Department of Neurosurgery, Medical College of Wisconsin, Milwaukee; 13 Department of Neurology, Medical College of Wisconsin, Milwaukee; 14 Department of Pediatric Neurosurgery, Mass General Hospital for Children, Boston, Massachusetts; 15 Department of Neurology & Rehabilitation Medicine, University of Cincinnati, Cincinnati, Ohio; 16 Department of Neurological Surgery, University of Utah, Salt Lake City; 17 Department of Laboratory Medicine and Pathology, University of Washington, Seattle; 18 Department of Anesthesiology, Duke University School of Medicine, Durham, North Carolina; 19 Department of Neurological Surgery, University of Washington, Seattle; 20 Department of Neurosurgery, Medical College of Wisconsin, Milwaukee; 21 Department of Anatomy and Neurobiology, Virginia Commonwealth University, Richmond; 22 Department of Neurological Surgery, University of Cincinnati, Cincinnati, Ohio; 23 Department of Neurological Surgery, University of Pittsburgh, Pittsburgh, Pennsylvania; 24 Department of Neurological Surgery, Goodman Campbell Brain and Spine, Indianapolis, Indiana; 25 Department of Psychology, University of Texas at Austin; 26 Department of Neurology, University of California, San Francisco; 27 Department of Physical Medicine and Rehabilitation, Massachusetts General Hospital, Boston

## Abstract

**Importance:**

Exposure to extracranial (EC) surgery early after traumatic brain injury (TBI) is associated with cognitive risks.

**Objective:**

To examine whether exposure to EC surgery during a TBI index admission is associated with worse outcomes at 1 year compared with no EC surgery.

**Design, Setting, and Participants:**

This was a retrospective secondary nested cohort study of the prospective, observational Transforming Research and Clinical Knowledge in TBI (TRACK-TBI) cohort study that enrolled participants from February 1, 2014, through August 31, 2018, at 18 US level I trauma centers. Participants aged 17 years or older who were admitted to an inpatient unit from the emergency department (ED) within 24 hours of trauma, had a known Glasgow Coma Scale (GCS) score and head computed tomography (CT) imaging, and did not undergo intracranial surgery were followed for up to 1 year after TBI and were analyzed for this study from July 25, 2023, to July 2, 2025.

**Exposure:**

Participants that underwent EC surgery during the index admission were compared with nonsurgical participants within the following injury subgroups: orthopedic trauma controls (OTCs), moderate-severe TBI (GCS 3-12), and computed tomography (CT) scan results that were positive (CT+) or negative (CT−) for acute intracranial findings along with a GCS score of 13 to 15.

**Main Outcomes and Measures:**

Brain injury–specific functional outcomes (Glasgow Outcome Scale–Extended [GOSE-TBI]), cognition (Trail Making Test [Trails] parts A and B), Disability Rating Scale (DRS), and Quality of Life After Brain Injury–Overall Scale (QOLIBRI-OS). A fixed-effects linear regression model with propensity weighting for missing outcome and group imbalance in baseline characteristics was used.

**Results:**

Of the 1835 participants, 1279 (70%) were male, with mean (SD) age of 42.2 (17.8) years; 1349 participants (74%) were nonsurgical and 486 (26%) underwent EC surgery. In the 1150 participants (63%) followed up at 1 year, after propensity weighting, patients undergoing EC surgery in both the CT+ TBI and moderate-severe TBI subgroups had significantly worse GOSE-TBI (*B*, −0.57 [95% CI, −0.92 to −0.22] and −1.25 [95% CI, −1.65 to −0.85], respectively), Trails part B (*B*, 22.7 [95% CI, 7.4-38.1] and 47.9 [95% CI, 27.0-68.8]), and DRS (*B*, 2.47 [95% CI, 1.30-3.64] and 3.53 [95% CI, 2.19-4.87]) scores compared with nonsurgical participants. QOLIBRI-OS was worse after EC surgery vs no EC surgery in the subgroup with moderate-severe TBI (*B*, −15.1 [95% CI, −24.3 to −5.9]). There was no association of EC surgery with outcomes in the OTC or CT− TBI subgroups. For example, GOSE-TBI was not associated with EC surgery in the CT− TBI subgroup (*B*, 0.02 [95% CI, −0.24 to 0.27]).

**Conclusions and Relevance:**

In this cohort study, early EC surgery was associated with adverse function, cognition, and disability after TBI rated as moderate-severe or with radiographic abnormalities on CT scan regardless of GCS at index admission but not after orthopedic trauma or CT− TBI. Further studies may help determine whether surgical timing or other interventions can improve the observed long-term deficits.

## Key Points

**Question:**

Are traumatic brain injury (TBI) outcomes at 1 year worse in individuals exposed to extracranial surgery early after trauma compared with people unexposed?

**Findings:**

In this multicenter cohort study of 1150 participants that had follow-up at 1 year, participants undergoing extracranial surgery early after TBI and those with acute intracranial findings on computed tomography had worse functional, cognitive, and disability outcomes compared with general orthopedic trauma patients or those with TBI with imaging negative for acute intracranial findings.

**Meaning:**

The results suggest that for patients with TBI-induced radiographic abnormalities, early extracranial surgery may generate additional neurotoxic mediators that act as secondary insults to an already injured brain.

## Teaser

This cohort study examines whether exposure to extracranial (EC) surgery during a traumatic brain injury index admission is associated with worse outcomes at 1 year compared with no EC surgery.

## Introduction

In population-based studies that do not control for traumatic brain injury (TBI), there is conflicting evidence as to whether surgery has neurotoxic effects that contribute to cognitive impairment. Surgery has been associated with increased neuronal injury biomarkers in some^1,2,3^ but not all studies.^4^ Also, to our knowledge, exposure to surgery has yet to demonstrate substantial, reproducible, and causal cognitive deficits at 1 year postinjury or beyond.^3,4,5,6,7,8,9,10,11,12,13,14,15,16,17,18,19^ These studies do not prove that exposure to surgery and anesthesia are safe for the brain but rather suggest that this exposure is minimally detrimental to the study populations. If general, pediatric, and geriatric populations are not at risk, this raises the question of which patients, if any,^20,21^ are vulnerable to neurotoxic sequelae from surgery.

Patients with a recent TBI are at risk of secondary insults, including physiologic disruptions from traumatic injuries, surgical stress responses, or other neurotoxic mediators.^22,23,24^ Up to 58% of TBIs occur simultaneously with peripheral, sometimes referred to as extracranial (EC), injuries that may require surgery.^25^ Surgeons, intensivists, and anesthesiologists are frequently tasked with weighing the risks and benefits of surgical interventions and their timing in patients with a recently injured brain. For example, an extremity injury has the potential to be permanently disabling or limb threatening, but aggressive repairs are not relevant if the brain is so severely injured that the central nervous system (CNS) is unable to control that extremity. Our group recently found that exposure to EC surgery shortly after TBI with radiographic abnormalities was associated with worse functional (Glasgow Outcome Scale–Extended [GOSE]) and neurocognitive (Trail Making Test part B [Trails B]) outcomes at 2 weeks and 6 months postinjury.^26^ These patient-relevant differences suggest that TBI of sufficient severity might be a risk factor for perioperative neurocognitive disorders.^27^ In the setting of TBI, there are limited data for follow-up beyond 6 months. The long-term sequalae and natural history of TBI are critically important at 1 year postinjury and beyond,^28,29,30,31^ but to our knowledge, they remain to be investigated in the context of perioperative neurocognitive disorders.^23,26,32^

This study using the Transforming Research and Clinical Knowledge in Traumatic Brain Injury (TRACK-TBI) database^33^ has several key differences compared with our group’s prior analysis of exposure to early EC surgery after TBI.^26^ First, the prior analysis controlled for computed tomography (CT) scan result categorization as positive (showing acute intracranial findings not counting skull fractures in isolation) or negative (not showing acute intracranial findings), whereas this study assessed the specific findings. Second, the prior analysis was conducted at 2 weeks and 6 months postinjury, whereas this study focused on 1 year postinjury. Third, targeted brain injury–specific outcomes using functional ability related to brain injury (GOSE-TBI) and neurocognitive outcomes (Trails A and B) were expanded to impairment, disability, and handicap (Disability Rating Scale [DRS]) and to health-related quality of life (HRQOL) using the Quality of Life After Brain Injury–Overall Scale (QOLIBRI-OS) in participants that underwent early EC surgery compared with nonsurgical patients. To mitigate the effects of deficits due to peripheral injuries, we included the peripheral Injury Severity Score (ISS) as a covariate. We hypothesized that exposure to surgery and, consequently, the perioperative period would be associated with long-term functional and cognitive deficits following TBI that impact disability and HRQOL.

## Methods

### Study Population and Design

This cohort study was a retrospective secondary nested analysis of the dataset from the prospective, observational TRACK-TBI cohort study. The TRACK-TBI study and this secondary analysis were completed in accordance with the ethical standards laid down in the 1964 Declaration of Helsinki,^34^ its later amendments, and all national laws and institutional regulations. The TRACK-TBI study was approved by the institutional review board (IRB) of each enrolling institution and led by the University of California, San Francisco. The Medical College of Wisconsin IRB approved this secondary data analysis. All participants or their legally authorized representatives gave written informed consent. This report follows the Strengthening the Reporting of Observational Studies in Epidemiology (STROBE) reporting guideline for cohort studies.

TRACK-TBI assessed participants in the following injury subgroups: CT imaging–negative (CT−) TBI with preserved Glasgow Coma Scale (GCS) score (13-15 on a scale of 3-15, with lower scores indicating less or worse responsiveness); CT imaging–positive (CT+) TBI with preserved GCS score (GCS 13-15), sometimes called mild TBI despite the associated morbidity and mortality^35^; moderate to severe TBI (GCS 3-12); or non-TBI orthopedic trauma controls (OTCs). Participants were enrolled from February 1, 2014, through August 31, 2018, at 18 level I trauma centers (eFigure 1 in Supplement 1) if they were seen within 24 hours of injury, were admitted to the hospital, had a known GCS score upon emergency department (ED) arrival, and had a clinically indicated head CT scan for all patients with a TBI (these were TRACK-TBI inclusion criteria).^36,37^ Scans were classified according to the National Institute of Neurological Disorders and Stroke Common Data Element Neuroimaging Working Group consensus recommendations.^38^ Participants with orthopedic injuries without evidence of head trauma were classified as OTCs. Exclusion criteria for TRACK-TBI enrollment included incarceration; pregnancy; nonsurvivable physical trauma, debilitating mental health disorders, or neurologic disease; or being non-English or non-Spanish speaking.

For this secondary analysis that was completed from July 25, 2023, to July 2, 2025, in participants who had follow-up 1 year after TBI, exclusion criteria were age younger than 17 years, incomplete surgical record, and any surgery not classified as extracranial (all surgical sites outside of the skull or intracranial vault). Additionally, participants were excluded if (1) any surgery was recorded as preinjury, (2) any surgery type was unknown, or (3) the only surgery was insertion of a tracheostomy tube, gastrostomy tube, or both. Surgeries were defined as early if they were conducted during the index admission only and were not tracked after discharge. Race and ethnicity were included in this study because these variables may be associated with outcomes after TBI; race categories were Black, White, and other (included Alaska Native or Inuit, Asian, Indian, mixed race, Native Hawaiian or Pacific Islander, and unknown; these were not further broken down due to small sample sizes), and ethnicity categories were Hispanic and non-Hispanic. The source of race and ethnicity (eg, medical records vs participant report) was not collected.

### Outcome Measures

Variables in the TRACK-TBI database were collected in accordance with the TBI Common Data Elements through direct assessment, medical records, and participant report, which have the potential to exhibit varying degrees of objectivity and susceptibility to measurement errors that could lead to inaccuracies.^39,40^ Functional outcomes were quantified for all injuries (GOSE-all; score range, 1-8, with higher scores indicating better function) and those attributed to brain injury only (GOSE-TBI).^41,42^ Cognitive outcomes included neuropsychological assessments of psychomotor processing speed and executive functioning or set shifting, measured using Trails A and B (part A, maximum 101 seconds; part B, maximum 301 seconds; lower scores indicate faster performance).^43,44,45,46^ Disability and HRQOL were assessed using the DRS (score range, 0-29; lower scores indicate less disability)^42,47^ and QOLIBRI-OS (score range, 0-100; higher scores indicate better QOL),^48,49,50^ respectively. Outcomes were assessed for GOSE-all, GOSE-TBI, Trails A, Trails B, DRS, and QOLIBRI-OS at 1 year after TBI in groups classified by injury and exposure to EC surgery. DRS and QOLIBRI-OS outcomes at 2 weeks and 6 months after TBI were included for completeness since they were not previously reported (eResults in Supplement 1). To avoid a selection bias that would skew the results toward the null hypothesis and to assess the need for an immortal time bias analysis, participants whose GOSE outcome was death (GOSE score = 1) within 2 weeks were excluded from the primary analysis (the eResults in Supplement 1 show models including those participants).

### Statistical Analysis

Groups were defined and finalized after a planned review of descriptive data prior to any analysis. Sample sizes limited further discrimination by TBI severity (especially in the GCS 9-12 group, which was therefore combined with GCS 3-8). Differences in demographic and clinical characteristics between the EC surgery and nonsurgical groups were assessed for statistical significance using Fisher exact tests for categorical variables and Mann-Whitney or Kruskal-Wallis tests for continuous and ordinal variables (Table 1). A fixed-effects linear regression model with propensity weighting to account for imbalances in baseline characteristics and unavailable data (treated as missing) evaluated the association between injury group, surgery groups, and injury × surgery interaction, with each clinical outcome and each time point modeled separately (eMethods in Supplement 1). Effect sizes were quantified as Cohen *d* values. Although residual diagnostics showed no substantial departures from normality, we also ran a sensitivity analysis using robust (sandwich) SEs as a more conservative approach. A 2-sided threshold of *P* < .05 defined statistical significance, with no adjustments for multiple comparisons. The boosted regression modeling was done using the Microsoft Windows (Microsoft Inc) version of the TWANG Shiny App software package developed by RAND Corporation. Regression modeling was carried out using SAS, version 9.4 (SAS Institute Inc), and other statistical analyses were conducted in SPSS Statistics for Windows, version 26 (IBM Corp).

**Table 1.** Clinical and Demographic Characteristics of the Study Participants

Characteristic	Injury group^a^								Standardized mean difference^c^			
	No surgery				Extracranial surgery^b^							
	Moderate-severe TBI, GCS 3-12 (n = 156)	CT+ TBI, GCS 13-15 (n = 546)^d^	CT− TBI, GCS 13-15 (n = 579)^e^	OTC (n = 68)	Moderate-severe TBI, GCS 3-12 (n = 98)	CT+ TBI, GCS 13-15 (n = 86)^d^	CT− TBI, GCS 13-15 (n = 181)^e^	OTC (n = 121)	Moderate-severe TBI	CT+ TBI^d^	CT− TBI^e^	OTC
GCS score, mean (SD)^f^	6.6 (3.3)	14.6 (0.6)	14.8 (0.5)	NA	5.5 (3.1)	14.5 (0.7)	14.8 (0.5)	NA	−0.17	0.05	0.03	−0.19
Age, mean (SD), y	41.8 (19.3)	47.9 (18.9)	37.8 (16.1)	45.2 (16.9)	36.6 (15.5)	50.2 (18.8)	38.2 (14.6)	40.5 (14.9)	−0.17	0.05	0.03	−0.19
Sex												
Female	30 (19)	162 (30)	217 (37)	26 (38)	22 (22)	20 (23)	49 (27)	30 (25)	0.02	−0.15	−0.27	−0.13
Male	126 (81)	384 (70)	362 (63)	42 (62)	76 (78)	66 (77)	132 (73)	91 (75)	−0.02	0.15	0.27	0.13
Educational level, mean (SD), y	12.9 (2.8)	13.5 (3.2)	13.2 (2.7)	14.0 (3.0)	12.4 (2.1)	13.6 (2.9)	12.9 (2.7)	13.3 (3.2)	−0.19	0.2	−0.07	−0.14
Race												
Black	31 (21)	59 (11)	123 (21)	9 (13)	17 (18)	10 (12)	28 (16)	22 (19)	0.02	0.01	−0.2	0.16
White	111 (74)	447 (83)	423 (73)	53 (79)	75 (79)	71 (86)	142 (80)	90 (76)	0.07	0.06	0.13	−0.14
Other^g^	9 (6)	32 (6)	31 (5)	5 (7)	3 (3)	2 (2)	8 (4)	6 (5)	−0.17	−0.23	0.04	−0.02
Unknown, No.	5	8	2	1	3	3	3	3	NA	NA	NA	NA
Ethnicity												
Hispanic	25 (17)	117 (22)	115 (20)	16 (24)	23 (24)	16 (19)	53 (30)	28 (24)	0.22	−0.14	0.12	−0.07
Non-Hispanic	124 (83)	420 (78)	460 (80)	51 (76)	73 (76)	67 (81)	126 (70)	91 (76)	−0.22	0.14	−0.12	0.07
Unknown, No.	7	9	4	1	2	3	2	2	NA	NA	NA	NA
Injury cause												
MVC, occupant	34 (22)	93 (17)	260 (45)	22 (32)	45 (46)	23 (27)	78 (43)	13 (11)	0.3	−0.03	−0.18	−0.41
MCC	20 (13)	40 (7)	47 (8)	8 (12)	22 (22)	13 (15)	23 (13)	20 (17)	0.05	0.06	0.08	−0.04
MVC, cyclist or pedestrian	18 (12)	90 (16)	63 (11)	7 (10)	13 (13)	18 (21)	26 (14)	7 (6)	−0.01	0.11	0.08	−0.09
Fall	46 (29)	211 (39)	122 (21)	16 (24)	11 (11)	22 (26)	27 (15)	37 (31)	−0.12	−0.02	−0.14	0.11
Assault	16 (10)	51 (9)	26 (4)	2 (3)	2 (2)	3 (3)	6 (3)	0	−0.16	−0.06	0.17	NA
Other or unknown	22 (14)	61 (11)	61 (11)	13 (19)	5 (5)	7 (8)	21 (12)	44 (36)	−0.45	−0.1	0.11	0.27
Lost consciousness	138 (95)	443 (87)	506 (90)	0	93 (100)	68 (84)	155 (89)	0	0.38	−0.09	0.00	NA
Posttraumatic amnesia	106 (96)	420 (83)	433 (78)	0	63 (91)	60 (91)	127 (79)	0	0.06	−0.04	−0.03	NA
Initial CT scan positive^d^	119 (82)	546 (100)	0	0	82 (87)	86 (100)	0	0	0.37	NA	NA	NA
ISS score, mean (SD)^h^												
Total	19.0 (11.1)	13.5 (7.4)	8.8 (6.5)	8.2 (5.0)	25.2 (12.5)	21.0 (10.2)	13.8 (8.5)	7.5 (6.3)	0.04	0.38	0.18	−0.17
Peripheral	5.1 (7.4)	3.2 (4.6)	5.4 (6.2)	8.1 (5.2)	12.8 (9.6)	11.2 (8.4)	10.5 (8.2)	7.3 (6.0)	0.24	0.42	0.03	−0.19
Maximum AIS peripheral score^i^												
Mean (SD)	1.5 (1.1)	1.2 (1.0)	1.6 (1.1)	2.4 (0.8)	2.6 (1.0)	2.5 (1.0)	2.4 (1.0)	2.3 (0.8)	0.29	0.40	0.00	−0.19
0	26 (17)	120 (23)	95 (18)	1 (2)	2 (2)	2 (2)	9 (5)	0				
1	67 (45)	241 (45)	176 (33)	5 (9)	9 (9)	12 (14)	20 (11)	16 (14)				
2	26 (17)	112 (21)	135 (25)	27 (47)	35 (36)	27 (32)	62 (35)	59 (51)				
3	21 (14)	51 (10)	116 (21)	22 (38)	35 (36)	33 (39)	74 (41)	35 (30)				
4	9 (6)	6 (1)	18 (3)	3 (5)	13 (13)	8 (10)	11 (6)	4 (3)				
5	0	1 (<1)	1 (<1)	0	3 (3)	2 (2)	3 (2)	2 (2)				
Shock index, mean (SD)^j^	0.71 (0.29)	0.63 (0.17)	0.65 (0.17)	0.62 (0.15)	0.79 (0.32)	0.67 (0.21)	0.68 (0.16)	0.62 (0.14)	0.21	−0.05	0.08	0.01
Hemoglobin, mean (SD), g/dL^j^	14.0 (1.7)	14.1 (1.6)	14.1 (1.7)	13.8 (1.6)	13.6 (2.0)	13.7 (1.7)	14.0 (1.7)	13.9 (1.6)	−0.27	−0.14	0.06	0.00
SpO_2_, mean (SD), %^j^	97.9 (4.0)	98.0 (2.1)	97.8 (2.9)	97.7 (2.3)	98.0 (3.5)	97.0 (3.7)	97.6 (3.4)	98.0 (2.2)	0.1	−0.09	−0.02	0.19
Base deficit, mean (SD), mEq/L^j^^,^^k^	3.6 (3.5)	2.6 (2.5)	2.0 (3.1)	0.6 (0.8)	4.4 (4.2)	4.8 (4.4)	2.7 (2.3)	1.6 (1.4)	0.08	0.47	0.13	0.83

Abbreviations: AIS, Abbreviated Injury Scale; CT, computed tomography; GCS, Glasgow Coma Scale; ISS, Injury Severity Score; MCC, motorcycle crash; MVC, motor vehicle crash; NA, not applicable; OTC, orthopedic trauma control; SpO_2_, oxygen saturation; TBI, traumatic brain injury.

SI conversion factor: To convert hemoglobin to g/L, multiply by 10.

^a^ Data are presented as number (percentage) of participants unless otherwise specified.

^b^ Refers to all surgeries outside of the skull, including maxillofacial procedures. All participants that underwent intracranial procedures (ie, craniotomy) were excluded.

^c^ Differences are for surgery comparisons weighted by inverse probability of surgery group membership.

^d^ Result of CT scan of the head was considered positive for acute intracranial findings not counting skull fractures in isolation (eResults in Supplement 1).

^e^ Result of CT scan of the head was considered negative for acute intracranial findings.

^f^ GCS range is 3 to 15, with lower scores indicating less or worse responsiveness.

^g^ Includes Alaska Native or Inuit, Asian, Indian, Mixed Race, Native Hawaiian or Pacific Islander, and unknown; these were not further broken down due to small sample sizes.

^h^ ISS score ranges from 0 to 75, with higher scores indicating greater injury. Peripheral indicates not the head or neck.

^i^ AIS score ranges from 0 to 6, with higher scores indicating greater injury. Peripheral indicates not the head or neck.

^j^ First assessment within 24 hours of admission for shock index, hemoglobin concentration, SpO_2_, and base deficit. Shock index is calculated as heart rate (beats per minute) divided by systolic blood pressure (mm Hg), with normal values in the range of 0.5 to 0.7 and higher values representing worse levels of shock.

^k^ Base deficit data were mostly missing (1167 of 1349 patients [87%] in the nonsurgical group and 362 of 486 [74%] in the surgical group).

## Results

### Participant Characteristics

After exclusions, there were 1835 participants (mean [SD] age, 42.2 [17.8] years; 556 females [30%], 1279 males [70%]), including 1646 with TBI (90%) and 189 OTCs (10%); 1349 participants (74%) were nonsurgical and 486 (26%) underwent EC surgery (Table 1 and eTable 1 in Supplement 1). A total of 299 participants (16%) were Black, 1412 (78%) were White, 96 (5%) were other race, and 28 had unknown race; 393 (22%) were Hispanic, 1412 (78%) were non-Hispanic, and 30 had unknown ethnicity. In all, 1150 participants (63%) had available 1-year outcomes (eTable 9 in Supplement 1). Within the TBI group, the sample size with available 1-year outcomes was 1032 participants (63%) for GOSE-all and GOSE-TBI, 734 (45%) for Trails A, 732 (44%) for Trails B, 993 (60%) for DRS, and 994 (60%) for QOLIBRI-OS (eFigure 1 in Supplement 1). Within the OTC group, the sample size with 1-year outcomes was 117 (62%) for GOSE-all, 84 (44%) for Trails A and Trails B, 113 (60%) for DRS, and 118 (62%) for QOLIBRI-OS (eFigure 1 in Supplement 1). The weighted nonsurgical and EC surgery groups were well balanced in age, educational level, race and ethnicity, injury cause, time from injury to ED admission, and ED disposition (Table 1 and eTable 1 in Supplement 1; injury groups combined are shown in eTable 2 in Supplement 1).

Participants undergoing EC surgery were first exposed to surgery within 15 days after TBI, with a mean (SD) of 3.0 (4.4) days and 3.7 (4.5) days in the CT+ and moderate-severe TBI groups, respectively (eTable 1 in Supplement 1). EC surgeries were primarily for extremity fracture (271 [56%]) (eTables 1 and 2 in Supplement 1). CT findings are reported by surgical exposure and injury subgroup (Table 1; eResults and eTables 1 and 2 in Supplement 1).

### One-Year Outcomes

**Figure 1.**
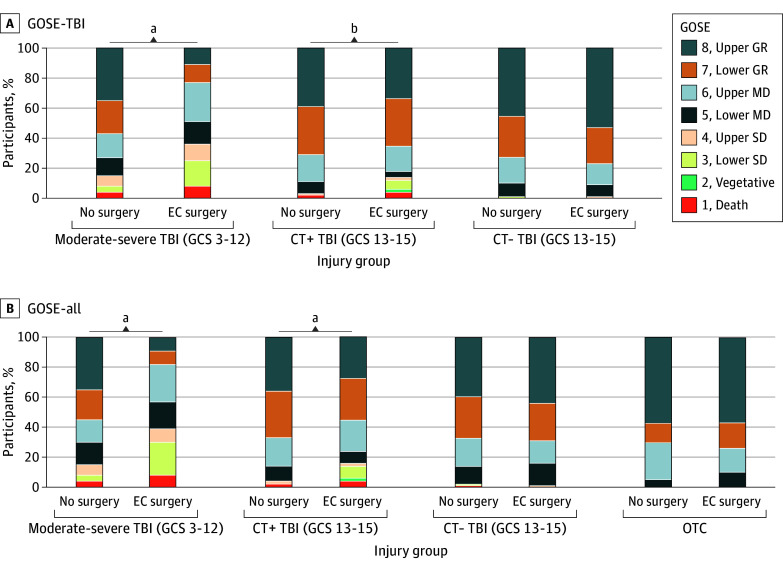
Functional Outcomes at 1 Year After Traumatic Brain Injury (TBI) Functional outcomes were quantified using the Glasgow Outcome Scale–Extended for brain injury (GOSE-TBI) and for all injuries (GOSE-all) at 1 year after TBI based on injury group and exposure to extracranial (EC) surgery without intracranial surgery, excluding deaths within 2 weeks of injury. Graphs show raw data. Significance is denoted for differences between nonsurgical and EC surgery groups in the interaction regression models in Table 2 with no adjustment for multiple comparisons. Additional data are provided in eTable 3 in Supplement 1. CT− indicates that CT imaging of the head was considered negative for acute intracranial findings; GR, good recovery; MD, moderate disability; OTC, orthopedic trauma control; SD, severe disability. ^a^*P* < .001. ^b^*P* = .002.

**Figure 2.**
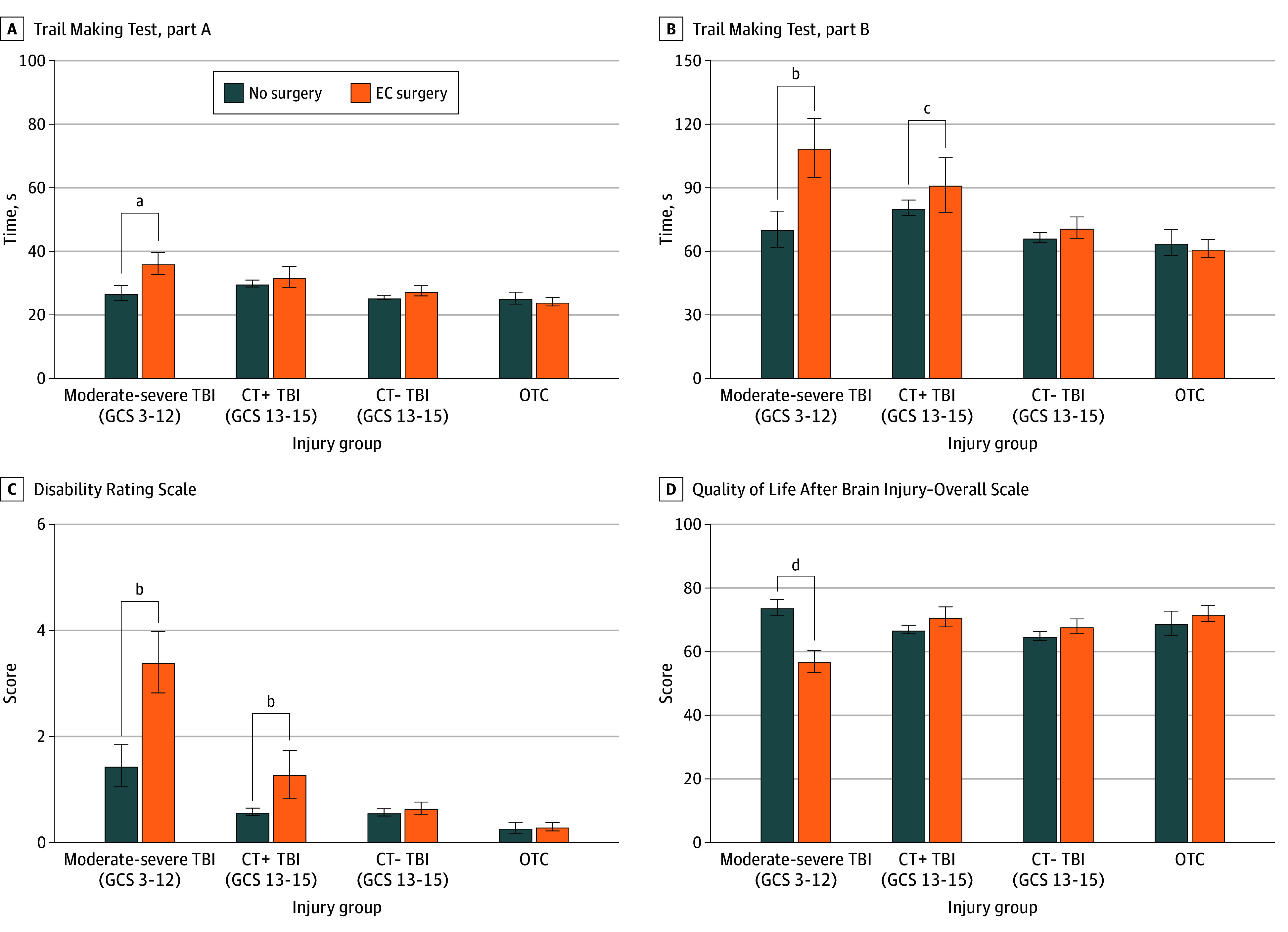
Outcomes for Cognition, Disability, and Quality of Life at 1 Year After Traumatic Brain Injury (TBI) A, Outcome was quantified as number of seconds to complete; maximum score, 101 seconds, and faster score is better. B, Outcome was quantified as number of seconds to complete; maximum score, 301 seconds, and faster score is better. C, Score range, 0-29, and lower score is better. D, Score range, 0-100, and higher score is better. Outcomes were classified by injury group and exposure to extracranial (EC) surgery without intracranial surgery. Graphs show raw unadjusted means, and whiskers indicate SEMs. Significance is denoted for differences between nonsurgical and EC surgery groups in the interaction regression models in Table 2 with no adjustment for multiple comparisons. CT− indicates CT scan of the head was considered negative for acute intracranial findings. ^a^*P* = .03. ^b^*P* < .001. ^c^*P* = .004. ^d^*P* = .001.

Participants exposed to EC surgery were compared with nonsurgical participants within each injury subgroup. Unadjusted outcomes are presented within each injury subgroup related to function (Figure 1), cognition (Figure 2A and B), disability (Figure 2C), and HRQOL (Figure 2D), with significance denoted by the regression models for the primary analysis such that comparisons reflect individuals who were living at 2 weeks postinjury (Table 2). Effect sizes were further quantified with Cohen *d* values (eTable 3 in Supplement 1). GOSE models including individuals who died within 2 weeks of injury yielded similar results (eResults, eFigure 2, and eTables 4 and 5 in Supplement 1).

**Table 2.** Regression Models for Functional, Neurocognitive, Disability, and Quality-of-Life Outcomes at 1 Year, Excluding Deaths Occurring Within 2 Weeks of Injury^a^

1-y Outcome	GOSE-all		GOSE-TBI		Trails part A		Trails part B		DRS		QOLIBRI-OS	
	*B* (95% CI)	*P* value	*B* (95% CI)	*P* value	*B* (95% CI)	*P* value	*B* (95% CI)	*P* value	*B* (95% CI)	*P* value	*B* (95% CI)	*P* value
Injury group^b^	NA	<.001	NA	<.001	NA	<.001	NA	<.001	NA	<.001	NA	.07
Moderate-severe TBI vs CT– TBI	−0.55 (−0.86 to −0.24)	<.001	−0.62 (−0.92 to −0.31)	<.001	3.04 (−1.29 to 7.37)	.17	7.7 (−7.2 to 22.7)	.31	2.64 (1.65 to 3.63)	<.001	4.01 (−2.51 to 10.53)	.23
CT+ TBI vs CT– TBI	−0.07 (−0.26 to 0.11)	.45	−0.13 (−0.31 to 0.06)	.17	3.68 (1.40 to 5.96)	.002	12.0 (4.1 to 19.9)	.003	0.02 (−0.57 to 0.62)	.94	1.10 (−2.71 to 4.90)	.57
OTC vs CT– TBI	0.24 (−0.15 to 0.63)	.23	NA	NA	−0.09 (−5.23 to 5.05)	.97	2.9 (−14.8 to 20.6)	.75	−0.83 (−2.10 to 0.44)	.20	4.72 (−3.39 to 12.83)	.25
Surgery × injury group^c^	NA	<.001	NA	<.001	NA	.34	NA	<.001	NA	<.001	NA	.02
EC surgery × moderate-severe TBI	−1.28 (−1.69 to −0.87)	<.001	−1.25 (−1.65 to −0.85)	<.001	6.64 (0.57 to 12.70)	.03	47.9 (27.0 to 68.8)	<.001	3.53 (2.19 to 4.87)	<.001	−15.1 (−24.3 to −5.9)	.001
EC surgery × CT+ TBI	−0.69 (−1.05 to −0.33)	<.001	−0.57 (−0.92 to −0.22)	.002	2.24 (−2.23 to 6.70)	.33	22.7 (7.4 to 38.1)	.004	2.47 (1.30 to 3.64)	<.001	0.72 (−7.20 to 8.64)	.86
EC surgery × CT– TBI	−0.02 (−0.28 to 0.24)	.90	0.02 (−0.24 to 0.27)	.90	1.47 (−1.65 to 4.60)	.36	3.4 (−7.3 to 14.2)	.53	0.19 (−0.62 to 1.00)	.65	1.03 (−4.12 to 6.17)	.70
EC surgery × OTC	0.02 (−0.43 to 0.48)	.92	NA	NA	−1.03 (−7.05 to 5.00)	.74	−6.0 (−26.7 to 14.8)	.57	0.10 (−1.39 to 1.60)	.89	1.75 (−7.78 to 11.28)	.72
Age, per 10-y increase	−0.07 (−0.11 to −0.02)	.003	−0.03 (−0.08 to 0.01)	.17	3.64 (3.09 to 4.19)	<.001	12.7 (10.8 to 14.6)	<.001	0.31 (0.17 to 0.46)	<.001	−1.58 (−2.50 to −0.66)	.001
Female vs male sex	−0.23 (−0.38 to −0.07)	.004	−0.21 (−0.37 to −0.05)	.01	−0.46 (−2.42 to 1.51)	.65	−5.0 (−11.8 to 1.7)	.15	0.37 (−0.13 to 0.87)	.14	−4.30 (−7.50 to −1.09)	.009
Race, with White as reference category	NA	.001	NA	.007	NA	<.001	NA	<.001	NA	.056	NA	.001
Black	−0.34 (−0.54 to −0.14)	.001	−0.25 (−0.46 to −0.04)	.02	7.97 (5.47 to 10.46)	<.001	32.3 (23.6 to 40.9)	<.001	0.60 (−0.05 to 1.24)	.07	−7.61 (−11.72 to −3.49)	<.001
Other or unknown	0.15 (−0.16 to 0.45)	.34	0.31 (−0.01 to 0.63)	.06	4.77 (1.09 to 8.45)	.01	12.9 (0.2 to 25.6)	.048	−0.67 (−1.65 to 0.30)	.17	2.82 (−3.42 to 9.05)	.38
Injury cause, with MVC as reference category	NA	.25	NA	.57	NA	.21	NA	.17	NA	.21	NA	.52
Fall	0.15 (−0.03 to 0.33)	.10	0.10 (−0.09 to 0.28)	.30	1.80 (−0.44 to 4.03)	.12	7.1 (−0.6 to 14.9)	.07	−0.51 (−1.08 to 0.06)	.08	1.10 (−2.60 to 4.79)	.56
Other or unknown	0.06 (−0.14 to 0.26)	.57	0.01 (−0.20 to 0.22)	.94	1.68 (−0.87 to 4.22)	.20	5.0 (−3.8 to 13.8)	.26	−0.08 (−0.72 to 0.57)	.82	−1.57 (−5.70 to 2.55)	.46
Educational level, per 4-y increase^d^	0.27 (0.17 to 0.37)	<.001	0.21 (0.10 to 0.31)	<.001	−6.43 (−7.72 to −5.14)	<.001	−25.8 (−30.2 to −21.3)	<.001	−0.56 (−0.88 to −0.23)	.001	4.57 (2.47 to 6.67)	<.001
ISS peripheral score, per 1-point increase	−0.01 (−0.02 to 0.01)	.30	0.01 (−0.01 to 0.02)	.33	0.16 (0.02 to 0.30)	.03	0.3 (−0.2 to 0.8)	.25	−0.03 (−0.07 to 0.00)	.07	0.11 (−0.13 to 0.34)	.38

Abbreviations: CT, computed tomography; DRS, Disability Rating Scale; EC, extracranial; GOSE, Glasgow Outcome Scale–Extended; ISS, Injury Severity Score; MVC, motor vehicle crash; NA, not applicable; OTC, orthopedic trauma control; QOLIBRI-OS, Quality of Life After Brain Injury–Overall Scale; TBI, traumatic brain injury; Trails, Trail Making Test.

^a^ A fixed-effects linear regression model evaluated the association between injury group, surgery group, and injury × surgery interaction within each clinical outcome and was propensity weighted for missing outcome and EC surgery group imbalance (age, sex, race, years of education, cause of injury, Glasgow Coma Scale score, non–head or neck ISS score, total ISS score, intensive care unit admission, and time from injury to admission).

^b^ The main effect sizes of the injury group presented reflect the outcome in those without EC surgery. The result of a CT scan of the head was considered either positive for acute intracranial findings not counting skull fractures (CT+) or negative for acute intracranial findings (CT–).

^c^ The main effect size of EC surgery without intracranial surgery in the model is reflected in the injury group × EC surgery (CT– TBI) contrasts. The result of a CT scan of the head was considered either positive for acute intracranial findings not counting skull fractures (CT+) or negative for acute intracranial findings (CT–).

^d^ Years of education is a complicated variable used as a proxy to account for multifactorial issues related to socioeconomic status and cognitive potential, which were partially confounded in this study, and included participants aged 17 years or older, since some participants had not yet completed their years of education.

Within the moderate-severe TBI subgroup, EC surgery compared with no surgery was associated with more TBI-related functional limitations (GOSE-TBI: *B*, −1.25 [95% CI, −1.65 to −0.85]; Cohen *d*, −0.92 [95% CI, −1.22 to −0.62]) (Figure 1), more injury-related functional limitations (GOSE-all: *B*, −1.28 [95% CI, −1.69 to −0.87]; Cohen *d*, −0.94 [95% CI, −1.25 to −0.63]), more disability (DRS: *B*, 3.53 [95% CI, 2.19-4.87]; Cohen *d*, 0.97 [95% CI, 0.60-1.34]), poorer processing speed (Trails A: *B*, 6.64 [95% CI, 0.57-12.70]; Cohen *d*, 0.42 [95% CI, −0.06 to 0.91]), poorer executive function (Trails B: *B*, 47.9 [95% CI, 27.0-68.8]; Cohen *d*, 1.01 [95% CI, 0.53-1.49]), and lower TBI-related quality of life (QOLIBRI-OS: *B*, −15.1 [95% CI, −24.3 to −5.9]; Cohen *d*, −0.59 [95% CI, −0.97 to −0.20]) (Figure 2 and eTables 6 and 7 in Supplement 1). Within the CT+ TBI subgroup, participants undergoing EC surgery had more TBI-related functional limitations (GOSE-TBI: *B*, −0.57 [95% CI, −0.92 to −0.22]; Cohen *d*, −0.42 [95% CI, −0.69 to −0.16]) (Figure 1), more injury-related functional limitations (GOSE-all: *B*, −0.69 [95% CI, −1.05 to −0.33]; Cohen *d*, −0.52 [95% CI, −0.79 to −0.25]), more disability (DRS: *B*, 2.47 [95% CI, 1.30-3.64]; Cohen *d*, 0.68 [95% CI, 0.36-1.00]), and poorer executive functioning (Trails B: *B*, 22.7 [95% CI, 7.4-38.1]; Cohen *d*, 0.53 [95% CI, 0.17-0.88]) (Figure 2 and eTables 6 and 7 in Supplement 1). There were no differences in TBI-related quality of life (QOLIBRI-OS: *B*, 0.72 [95% CI, −7.20 to 8.64]; Cohen *d*, 0.03 [95% CI, −0.30 to 0.36]) or processing speed (Trails A: *B*, 2.24 [95% CI, −2.23 to 6.70]; Cohen *d*, 0.42 [95% CI, −0.06 to 0.91]). Within the CT− TBI and OTC subgroups, EC surgery was not associated with TBI-related functional limitations (GOSE-TBI: *B*, 0.02 [95% CI, −0.24 to 0.27]; Cohen *d*, 0.01 [95% CI, −0.18 to 0.20]) or injury-related functional limitations (GOSE-all: *B*, −0.02 [95% CI, −0.28 to 0.24]; Cohen *d*, −0.01 [95% CI, −0.21 to 0.18] for the CT− TBI subgoup; and *B*, 0.02 [95% CI, −0.43 to 0.48]; Cohen *d*, 0.03 [95% CI, −0.32 to 0.37] for the OTC subgroup) (Figure 1) or with disability (DRS), executive function (Trails B), processing speed (Trails A), or TBI-related quality of life (QOLIBRI-OS) (Figure 2 and eTables 6 and 7 in Supplement 1).

Where model-based analyses suggested statistically significant group differences, these effects were attenuated when using robust SEs (eTable 8 in Supplement 1). Specifically, differences between the CT+ TBI groups remained significant for GOSE-all, Trails A, and Trails B but were no longer significant for GOSE-TBI (*B*, −0.57 [95% CI, −1.19 to 0.04]; *P* = .07) or DRS (*B*, 2.47 [95% CI, −0.08 to 5.02]; *P* = .06).

## Discussion

The results of this study demonstrate an association between early EC surgery after TBI and worse functional, cognitive, and disability outcomes at 1 year postinjury for participants with CT+ TBI or with TBI classified as moderate-severe. Those in the moderate-severe TBI subgroup additionally reported poorer quality-of-life outcomes at 1 year. These findings are consistent with our group’s previously published outcomes at 2 weeks and 6 months after TBI^26^ and suggest persistent deficits for at least 1 year. These effect sizes were clinically meaningful deficits in function (GOSE scores that were lower [ie, worse] by 0.57 to 1.25 points, where 1-point changes correspond to major changes in independence—for example, being dependent vs independent at home; working restricted hours or duties vs being fully back to preinjury work level), cognition (Trails B times that were longer [ie, worse] by 22.7 to 47.9 seconds when completing an executive function task that would normally take approximately 70 seconds), and disability (DRS scores that were higher [ie, worse] by 2.47 to 3.53 points, where a 1-point difference is considered clinically important).^51^ Further studies are urgently needed, as there are currently limited data to accurately assess the risk-to-benefit ratio of EC surgery shortly following TBI. The clinical concern is that later operations may increase nonunion,^52^ the number of surgeries necessary, or surgical site infections, which have been associated with long-term functional disability.^53,54^

Although functional outcomes (GOSE) have a dose-response association with TBI severity,^55^ prior studies have infrequently reported brain injury–specific functional outcomes (GOSE-TBI), which are essential to quantify the risk to the brain in the setting of multiple trauma.^29,30,31,55^ These data suggest that early EC surgery after TBI and any associated complications may represent a secondary insult to the brain that worsens functional outcomes (GOSE-all and GOSE-TBI) for at least a year. In the CT+ and moderate-severe TBI groups, EC surgery had comparable outcomes for GOSE-all and GOSE-TBI scores, suggesting that the functional deficits are primarily driven by the brain injury rather than the peripheral traumatic injuries (Figure 1 and Table 2). Ongoing trials are examining the long-term effect of surgery on the brain in non-TBI geriatric^56^ and pediatric^57,58,59^ populations, but studies are lacking and urgently needed in patients with TBI undergoing EC surgery. The optimal anesthesia for procedures^60^ or sedation for intensive care unit management also merit further investigation.^61^

Performance on the neurocognitive tests at 1 year after TBI paralleled our group’s previously published findings at 6 months.^26^ Trails B performance was worse after EC surgery in the CT+ and moderate-severe TBI groups but not different in the CT− TBI or OTC groups. Trails A performance did not differ between EC surgery and nonsurgical groups, except in the moderate-severe TBI subgroup, suggesting that EC surgery impacted executive functioning (set shifting) differentially compared with psychomotor processing speed. These findings are consistent with other studies reporting that the orthopedic trauma surgery population is not at risk of delirium from exposure to general vs regional anesthesia but rather from the acute trauma or surgery.^10,11,62^

We found that patient-oriented assessments of disability and HRQOL were worse for participants exposed to EC surgery compared with nonsurgical counterparts in the moderate-severe TBI groups. In the CT+ TBI groups, there was worse disability but no change in HRQOL. These patterns are generally congruent with the changes we observed in functional and cognitive outcomes, making it tempting to postulate that the cause is a brain-specific secondary insult. HRQOL following TBI, quantified with QOLIBRI-OS, is often rated satisfactory regardless of injury severity and functional limitations.^63,64^ This is relevant to our findings that patients with moderate-severe TBI exposed to EC surgery had worse QOLIBRI-OS scores. One possible explanation is that there is a detrimental, synergistic interaction between worsened brain injury and the ability to compensate for deficits from peripheral injuries. If true, this may suggest that the body cannot compensate for a peripheral injury in the setting of a concurrent or recent TBI of sufficient severity, possibly due to lost CNS plasticity.^65,66^

Timing of surgical interventions is an important clinical decision, especially early after TBI when the brain is vulnerable to secondary insults.^67^ In this study, participants were first exposed to EC surgery within 15 days after TBI,^26^ with a mean (SD) of 3.0 (4.4) and 3.7 (4.5) days in the CT+ and moderate-severe TBI groups, respectively (eTable 2 in Supplement 1). This timing is similar to that reported by Zheng et al,^68^ who compared early (defined as within 24 hours of injury) vs late extremity fixation. The median timing of surgery in the late group was 7.1 days (IQR, 3.7-12 days; Hong Fu, MD, Department of Anesthesiology, Chongqing University Central Hospital, Chongqing, China; email communication, April 16, 2024). In that study, GOSE did not differ between early vs late fixation, but there was no nonsurgical group. Most studies comparing early vs late fracture fixation lack a nonsurgical comparator group and have similarly found no difference in neurologic outcomes based on time to surgical intervention.^52,68,69^ These studies are limited because the operative interventions occurred at less than 7 days^69^ or 14 days^52^ postinjury and, therefore, all patients would have been exposed to surgery during a time that could worsen brain injury outcomes, according to the findings of this study. Additionally, the neurologic outcomes assessed were acute (during the index admission or at discharge),^52,69^ which do not accurately prognosticate patient outcomes at 6 months or 1 to 7 years postinjury.^30,31^ Investigations with nonsurgical comparators and brain injury–specific outcomes are rare, underpowered, and likely to include patients with CT− TBI,^32^ a subgroup not at risk in this study. Taken together with our data, these findings suggest that patients are potentially in a critical window of vulnerability within hours until weeks after TBI, but the exact timing is unknown. This timing is critically important in determining if damage-control surgical approaches (eg, external fixation) are a viable clinical decision and warrants further investigation.^70,71^ The pathophysiologic mechanisms underlying this vulnerability are unknown, but the roles of inflammation, cell signaling, and blood-brain barrier permeability have been investigated in TBI.^72,73,74,75^ Also, it remains to be determined if EC surgery is a secondary insult in other CNS disorders, such as anoxic injuries due to cardiac arrest or hemorrhagic, ischemic, toxic-metabolic, infectious, or spinal cord injuries.^76^ Furthermore, the underlying pathophysiologic mechanisms responsible for secondary insults due to surgical stress responses may also have a role in disorders that trigger immune or inflammatory responses, such as sepsis.^77,78^ If so, there are a host of other potential secondary insults (eg, postoperative complications, infections, or hemorrhage) that could compromise brain injury or recovery when the CNS is in a vulnerable state.

In the near term, individualizing care might be possible if serologic or imaging-based biomarkers can define phenotypes that predict vulnerability to secondary insults.^79,80^ Long term, this could revolutionize care for patients with TBI and potentially dementia or other neurologic disorders if neuroprotective therapies to prevent secondary brain injury could be delivered such that elective, urgent, and emergent operations could proceed without delay or risk. However, some of the neurocognitive issues uncovered may relate not only to the intraoperative events of exposure to surgery and anesthesia but also to postoperative events that require heightened detection, therapy, and rescue. Achieving these goals necessitates collaboration of multidisciplinary and interprofessional teams to further understand the complex pathophysiologic mechanisms of patients with brain injury in the perioperative period. In the meantime, coordinating care of these patients across multiple specialties is essential to optimize outcomes.

### Limitations

This study has several limitations, as described in our group’s previously published analysis.^26^ Foremost is the concern for confounding by indication. For example, it is possible that there were more severe traumas or brain injuries or an interaction with peripheral injury or multiple trauma that directly influenced the surgical group such that there were worse outcomes despite similar clinical presentations.^81^ Second, there were unquantified variables, such as delirium, which has been associated with long-term cognitive impairment in some^82,83,84^ but not all studies.^85,86,87,88^ Third, peripheral or multiple-traumatic insults were treated as covariates and therefore adjusted for the peripheral ISS in these models, but EC surgery was the variable of interest. Therefore, the association of peripheral ISS with outcomes was not reported in the TBI severity subgroups. Fourth, individual patient trajectories were not interrogated, but these vary for TBI-induced insomnia^89,90^ and neuropsychiatric symptoms.^91^ Fifth, some caution is warranted in interpreting these findings because the associations were attenuated where outcome distribution and weighting may have introduced unmodeled variance. Sixth, this observational study demonstrated an association, which cannot be misconstrued to suggest causality or identify a pathophysiologic mechanism. As such, the findings presented herein require replication using other observational datasets. If reproduced, these results should be tested in preclinical models to demonstrate causality. Messaging of these results, especially to the general population, is critically important because misperceptions could have serious implications.^92^

An important limitation of our analytic approach is that inverse probability weighting, while useful for addressing covariate imbalance and missing data, can induce dependence among observations, potentially affecting the accuracy of SE estimates. Although our primary analyses used model-based SEs under the assumption of independent and homoscedastic residuals, we conducted sensitivity analyses using robust (sandwich) SEs to account for this potential dependence. These analyses showed that for outcomes with approximately gaussian distributions, results were largely consistent. However, for nonnormally distributed outcomes such as GOSE, robust SEs were wider and CIs more conservative, suggesting some sensitivity to model assumptions. We acknowledge this as a source of uncertainty and have interpreted the results accordingly.

## Conclusions

In this multicenter cohort study, we found that exposure to EC surgery early after TBI was associated with worse function, cognition, and disability at 1 year in persons with TBI rated as moderate-severe and in the subgroup with acute intracranial findings on neuroimaging that are paradoxically classified as mild TBI using GCS.^35^ These findings suggest that imaging might be more valuable in assessing vulnerability to secondary insults than clinical assessments of alertness and responsiveness. This association was not observed in participants with general orthopedic trauma or imaging-negative TBI populations. Further studies are necessary to determine the pathophysiologic mechanisms responsible for these findings so that secondary insults can be avoided or prevented. It is especially important to discover if surgical timing or other interventions can improve the observed long-term deficits.

## Notes

Supplement 1. **eFigure 1.** Recruitment and Retention Flowchart **eMethods.** Propensity Weighting and Regression Models **eResults.** Outcomes at 1 Year Postinjury Including Individuals Who Died Within 2 Weeks of Injury and Outcomes at 2 Weeks and 6 Months Postinjury That Were Not Previously Reported **eFigure 2.** Functional Outcomes at 1 Year After Traumatic Brain Injury Including Deaths Within 2 Weeks of Injury **eTable 1.** Clinical and Demographic Characteristics of the Study Participants Separated by Traumatic Brain Injury (TBI) and Orthopedic Trauma Control Subgroups and Exposure to Extracranial Surgery **eTable 2.** Clinical and Demographic Characteristics of Study Participants in the No Surgery and Extracranial Surgery Groups **eTable 3.** Standardized Cohen d Effect Sizes From Regression Models for Functional, Neurocognitive, Disability, and Quality-of-Life Outcomes at 1 Year, Excluding Deaths Occurring Within 2 Weeks of Injury **eTable 4.** Functional outcome assessments among TBI and OTC Participants: Glasgow Outcome Scale-Extended (GOSE) **eTable 5.** Regression Models for Functional Outcomes at 1 Year, Including Deaths Occurring Within 2 Weeks of Injury **eTable 6.** Neurocognitive, Disability, and Quality-of-Life Outcome Assessments Among TBI and OTC Participants at 2 Weeks, 6 Months, and 1 Year After TBI **eTable 7.** Regression Models for Disability and Quality-of-Life Outcomes at 2 Weeks and 6 Months, Excluding Deaths Occurring Within 2 Weeks of Injury **eTable 8.** Regression Models for Functional, Neurocognitive, Disability, and Quality-of-Life Outcomes at 1 Year, Excluding Deaths Occurring Within 2 Weeks of Injury, With Robust Estimation of SEs **eTable 9.** Participants Lost to Follow-up at 1 Year After Trauma

Supplement 2.
Data Sharing Statement
