# Supplementary material for: One-Year Outcomes After Traumatic Brain Injury and Early Extracranial Surgery in the TRACK-TBI Study
Source: JAMA Netw Open. 2025 Oct 10;8(10):e2537271. doi: 10.1001/jamanetworkopen.2025.37271 (PMC12514633; doi:10.1001/jamanetworkopen.2025.37271)
Supplement: Supplement 1. — eFigure 1. Recruitment and Retention Flowchart eMethods. Propensity Weighting and Regression Models eResults. Outcomes at 1 Year Postinjury Including Individuals Who Died Within 2 Weeks of Injury and Outcomes at 2 Weeks and 6 Months Postinjury That Were Not Previously Reported eFigure 2. Functional Outcomes at 1 Year After Traumatic Brain Injury Including Deaths Within 2 Weeks of Injury eTable 1. Clinical and Demographic Characteristics of the Study Participants Separated by Traumatic Brain Injury (TBI) and Orthopedic Trauma Control Subgroups and Exposure to Extracranial Surgery eTable 2. Clinical and Demographic Characteristics of Study Participants in the No Surgery and Extracranial Surgery Groups eTable 3. Standardized Cohen d Effect Sizes From Regression Models for Functional, Neurocognitive, Disability, and Quality-of-Life Outcomes at 1 Year, Excluding Deaths Occurring Within 2 Weeks of Injury eTable 4. Functional outcome assessments among TBI and OTC Participants: Glasgow Outcome Scale-Extended (GOSE) eTable 5. Regression Models for Functional Outcomes at 1 Year, Including Deaths Occurring Within 2 Weeks of Injury eTable 6. Neurocognitive, Disability, and Quality-of-Life Outcome Assessments Among TBI and OTC Participants at 2 Weeks, 6 Months, and 1 Year After TBI eTable 7. Regression Models for Disability and Quality-of-Life Outcomes at 2 Weeks and 6 Months, Excluding Deaths Occurring Within 2 Weeks of Injury eTable 8. Regression Models for Functional, Neurocognitive, Disability, and Quality-of-Life Outcomes at 1 Year, Excluding Deaths Occurring Within 2 Weeks of Injury, With Robust Estimation of SEs eTable 9. Participants Lost to Follow-up at 1 Year After Trauma [file jamanetwopen-e2537271-s001.pdf]

Supplementary Online Content

Roberts CJ, Maiga AW, Barber J, et al; Transforming Clinical Research and Knowledge in TBI (TRACK-TBI) Investigators. One-year outcomes after traumatic brain injury and early extracranial surgery in the TRACK-TBI study. *JAMA Netw Open*. 2025;8(10):e2537271. doi:10.1001/jamanetworkopen.2025.37271

eFigure 1. Recruitment and Retention Flowchart
eMethods. Propensity Weighting and Regression Models
eResults. Outcomes at 1 Year Postinjury Including Individuals Who Died Within 2 Weeks of Injury and Outcomes at 2 Weeks and 6 Months Postinjury That Were Not Previously Reported
eFigure 2. Functional Outcomes at 1 Year After Traumatic Brain Injury Including Deaths Within 2 Weeks of Injury
eTable 1. Clinical and Demographic Characteristics of the Study Participants Separated by Traumatic Brain Injury (TBI) and Orthopedic Trauma Control Subgroups and Exposure to Extracranial Surgery
eTable 2. Clinical and Demographic Characteristics of Study Participants in the No Surgery and Extracranial Surgery Groups
eTable 3. Standardized Cohen *d* Effect Sizes From Regression Models for Functional, Neurocognitive, Disability, and Quality-of-Life Outcomes at 1 Year, Excluding Deaths Occurring Within 2 Weeks of Injury
eTable 4. Functional outcome assessments among TBI and OTC Participants: Glasgow Outcome Scale-Extended (GOSE)
eTable 5. Regression Models for Functional Outcomes at 1 Year, Including Deaths Occurring Within 2 Weeks of Injury
eTable 6. Neurocognitive, Disability, and Quality-of-Life Outcome Assessments Among TBI and OTC Participants at 2 Weeks, 6 Months, and 1 Year After TBI
eTable 7. Regression Models for Disability and Quality-of-Life Outcomes at 2 Weeks and 6 Months, Excluding Deaths Occurring Within 2 Weeks of Injury
eTable 8. Regression Models for Functional, Neurocognitive, Disability, and Quality-of-Life Outcomes at 1 Year, Excluding Deaths Occurring Within 2 Weeks of Injury, With Robust Estimation of SEs
eTable 9. Participants Lost to Follow-up at 1 Year After Trauma

This supplementary material has been provided by the authors to give readers additional information about their work.

© 2025 Roberts CJ et al. *JAMA Network Open*.

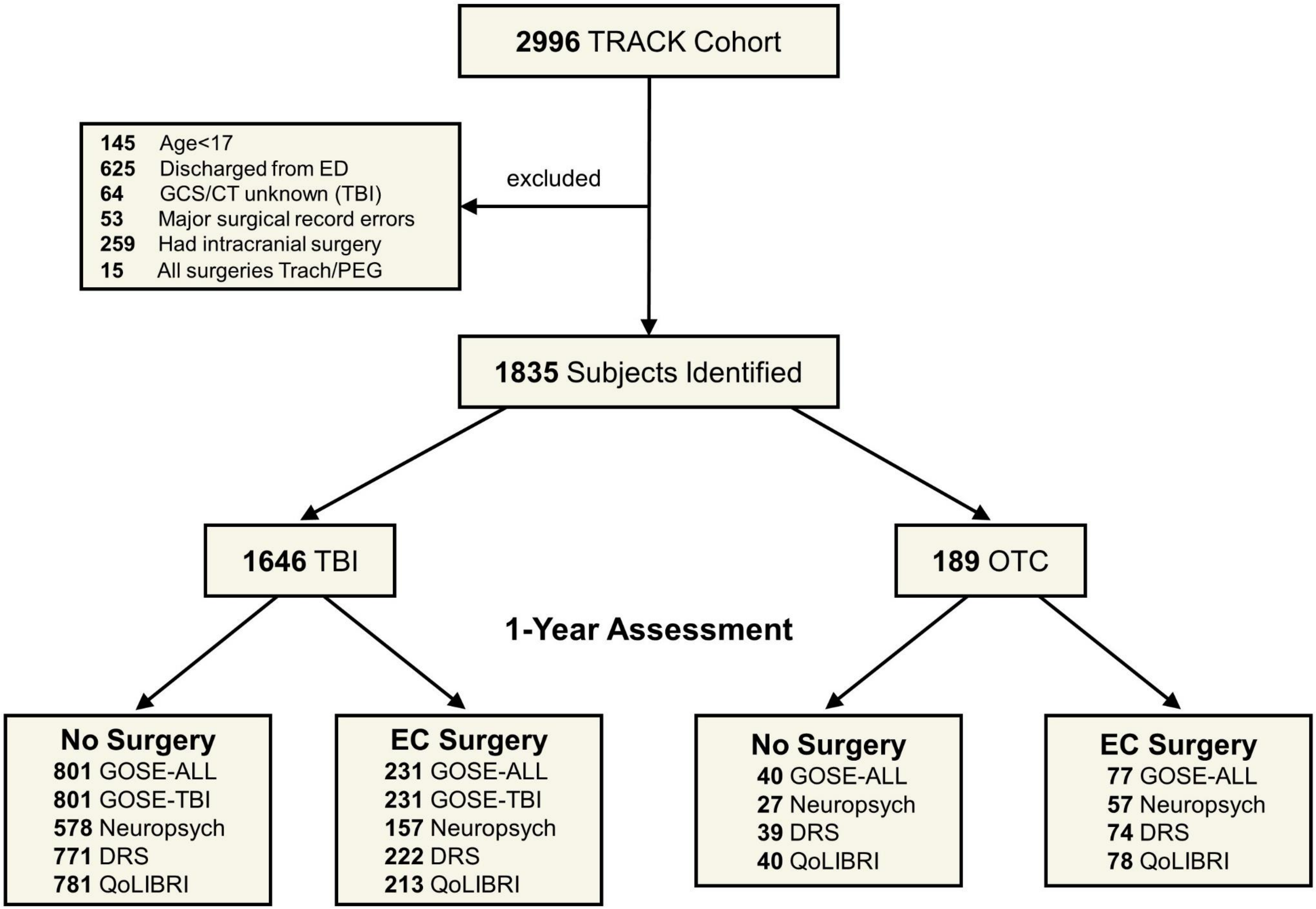

eFigure 1. Recruitment and Retention Flowchart. Abbreviations: traumatic brain injury (TBI), orthopedic trauma control (OTC), extracranial (EC) surgery, Glasgow Outcome Scale–Extended for all injuries (GOSE-ALL) and brain injury (GOSE-TBI), neuropsychological tests (Neuropsych), tracheostomy tube (trach), percutaneous endoscopic gastrostomy (PEG), and emergency department (ED).

© 2025 Roberts CJ et al. *JAMA Network Open*.

eMethods.

The main analysis implemented a fixed effects linear regression model. A primary interest was in planned contrasts for non-surgical versus EC Surgery within each injury subgroup (OTC, CT-TBI, CT+TBI, and m/sTBI). Subjects were included in the EC Surgery group if they had one or more surgeries during the index admission with rates of multiple surgeries ranging from 24 – 51% (**eTable 2**) with lower rates in the OTC and CT-TBI subgroups and higher rates in the CT+TBI and m/sTBI subgroups. Inverse probability weighting was used in the models to try to achieve parity between the 2 surgery groups on key characteristics listed in Table 1 (age, sex, race, years of education, cause of injury, GCS, Peripheral ISS score, total ISS score, ICU admission, and time from injury to admission), and to help account for any potential bias due to unassessed outcomes. When an outcome was unavailable due to deaths and untestable conditions it was treated as missing using inverse probability weighting. In brief, inverse probability weighting more heavily weights data from groups that were underrepresented in follow-up data, which allows findings to be extrapolated to the full enrolled sample. To this end, boosted binary regression models were constructed for surgery group membership and for each outcome at each time point. Predicted probabilities resulting from each of the 10 models were inverted and rescaled to form standardized weights. The 10 models were for 1) GOSE-TBI at 1 year; 2) GOSE-ALL at 1 year; 3) Trails A at 1 year; 4) Trails B at 1 year; 5) DRS at 1 year; 6) QoLIBRI-OS at 1 year; 7) DRS at 2 weeks; 8) QoLIBRI-OS at 2 weeks; 9) DRS at 6 months; and 10) QoLIBRI-OS at 6 months. Then the outcome and surgery-group weights were combined for the regression modeling by multiplying and doing a final rescaling, so the sum equaled the number of cases with the measure completed. Another planned analysis prior to group finalization and analysis was to analyze the above interaction regression models without exclusion of deaths within 2-weeks of TBI. Deaths within 2-weeks of TBI were excluded from the primary analysis so that non-survivable injuries, withdrawal of care, or futility did not bias the results towards no difference between the EC Surgery and non-surgical groups. The inclusion of results for participants that died within 2-weeks post-injury only influenced the models for GOSE-TBI and GOSE-ALL and not the other models because unavailable data was treated as missing. A Cohen's d standardized effect size was estimated by dividing the regression

© 2025 Roberts CJ et al. *JAMA Network Open*.

coefficient by the standard deviation of the model residuals (eTable 4, 6, and 8), where values of 0.2, 0.5, and 0.8 are considered small, medium, and large effects, respectively.

The boosted regression modeling was done using the Windows version of the TWANG Shiny App software package developed by RAND Corporation (June 2020). Regression modeling was carried out using SAS statistical software version 9.4 (SAS Institute, Cary, NC), and other statistical analyses were conducted in SPSS Statistics for Windows, version 26 (IBM Corporation).

CT scans were classified as positive if found to have an acute intracranial finding on the initial imaging during the index admission according to the NINDS Common Data Element Neuroimaging Working Group consensus recommendations. Findings tracked included, but were not limited to subarachnoid hemorrhage, subdural hematoma, epidural hematoma, cerebral contusion, intraventricular hemorrhage, shear, intracerebral hematoma, pneumocephalus, edema, midline shift, herniation, infarction, hydrocephalus, and vascular injury. Notably, skull fractures were tracked, but were not considered to be an intracranial abnormality to remain consistent with prior publications from the TRACK-TBI database. Statistical significance is reported for injury subgroup differences using a weighted comparison between non-surgical and EC Surgery groups with positive findings subdivided by variations of positive intracranial abnormalities (i.e. yes positive versus yes with no other intracranial injuries, etc), but no p values were meaningfully different from results with positive findings collapsed.

© 2025 Roberts CJ et al. *JAMA Network Open*.

eResults.

Outcomes at 1 Year Postinjury Including Individuals Who Died Within 2 Weeks of Injury

In planned comparisons including participants with deaths within 2 weeks of injury, subgroups of injury severity were compared between groups that underwent EC-Surgery and their non-surgery counterparts. Raw data for GOSE-TBI and GOSE-ALL (**eFigure 2; eTable 5**) were analyzed in separate models (**eTable 6**). Within the m/sTBI group (**eTable 6**), EC-surgery was associated with more TBI-related functional limitations (GOSE-TBI B = -0.73 [95%CI -1.21 to -0.24]; Cohen's d = -0.49), and more injury-related functional limitations (GOSE-ALL B = -0.78 [95%CI -1.26 to -0.31]; d = -0.54). Results for disability (DRS), processing speed (Trails A), executive functioning (Trails B), and TBI-related quality of life (QoLIBRI-OS) are identical when including individuals who died within 2 weeks of injury because unavailable data were treated as missing in all analyses.

Within the CT+TBI group (**eTable 6**), EC-surgery was associated with more TBI-related functional limitations (GOSE-TBI B = -0.57 [95%CI -1.00 to -0.13]; Cohen's d = -0.38), and more injury-related functional limitations (GOSE-ALL B = -0.67 [95%CI -1.09 to -0.24]; d = -0.46). Within the CT-TBI and OTC subgroups, EC-surgery was not associated with TBI-related functional limitations (GOSE-TBI) or injury-related functional limitations (GOSE-ALL).

Imaging results on initial presentation

Head CT scan findings at the time of the initial presentation during the index admission are reported by surgical exposure and injury subgroup (**eTable 2**) and surgical exposure alone (**eTable 3**). CT scans positive for acute intracranial findings were not significantly different based on surgical exposure within any of the injury subgroups (**eTable 2**).

Outcomes at 2 weeks and 6 months postinjury

Outcomes for disability (DRS) and TBI-related quality of life (QoLIBRI-OS) at 2-weeks and 6-months post-injury were not previously published and therefore are included here for completeness (**eTable 7; eTable 8)**. In the m/sTBI group, EC Surgery as associated with more disability at 2 weeks (DRS B = 4.33 [95%CI 2.52 to

© 2025 Roberts CJ et al. *JAMA Network Open*.

6.15]; d = 0.75) and 6 months (DRS B = 3.93 [95%CI 2.60 to 5.27]; d =1.04) and lower TBI-related quality of life at 2 weeks (QoLIBRI-OS B = -11.27 [95%CI -21.38 to -1.17]; d = -0.49) and 6 months (QoLIBRI-OS B = -10.50 [95%CI -19.34 to -1.66]; d = -0.43). Results were previously published for GOSE-TBI, GOSE-ALL, Trails A and B at 2 weeks and 6 months with separate models that excluded and included individuals who died within 2 weeks of injury. In the CT+TBI group, EC Surgery as associated with more disability at 2 weeks (DRS B = 5.56 [95%CI 3.95 to 7.17]; d = 0.97) and 6 months (DRS B = 2.55 [95%CI 1.36 to 3.74]; d =0.67). In the CT+TBI group, HRQoL was not different between EC Surgery and non-surgical groups at 2 weeks or 6 months. At 2 weeks and 6 months, participants demonstrated a similar pattern of differences between EC-Surgery and non-surgical groups (**eTable 7; eTable 8**) as was seen at 1 year.

As expected, there was loss to follow-up at 1-year after the index admission for TBI and OTC participants. There was not a differential loss to follow-up in the groups exposed to EC Surgery compared to No surgery groups within the OTC or any of the TBI severity subgroups (**eTable 10**).

© 2025 Roberts CJ et al. *JAMA Network Open*.

eFigure 2. Functional Outcomes at 1 Year after Traumatic Brain Injury including deaths within 2 weeks of injury. Functional outcomes quantified using Glasgow Outcome Scale–Extended for brain injury (GOSE-TBI) and all injuries (GOSE-ALL) at 1 year after TBI based on injury group and exposure to extracranial surgery. **A)** GOSE-TBI; **B)** GOSE-ALL. Graphs are raw data with sample sizes displayed at bottom of each column. Significance denoted by *p<0.05; **p<0.01; ***p<0.001; for differences between non-surgical and EC Surgery groups in the interaction regression models in eTable 5 with no adjustment for multiple comparisons. Additional data provided in eTable 4 for total sample sizes and Mean (SD). Abbreviations: good recovery (GR), moderate disability (MD), severe disability (SD), negative computed tomography scan TBI (-CT scan; CT-TBI), positive CT scan TBI (+CT scan; CT+TBI), moderate to severe TBI (m/sTBI), orthopedic trauma control (OTC), extracranial (EC) surgery.

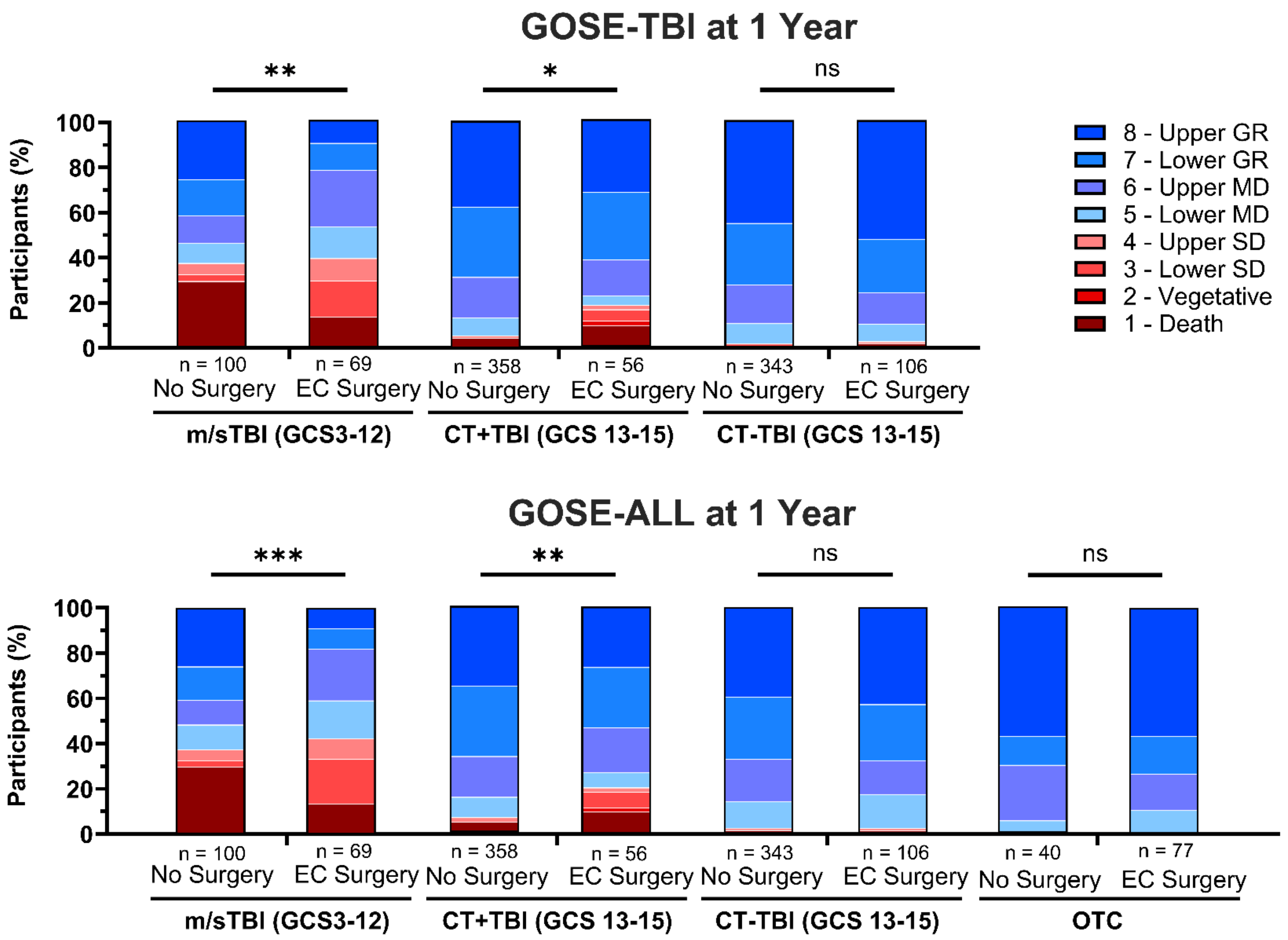

© 2025 Roberts CJ et al. *JAMA Network Open*.

eTable 1. Clinical and Demographic Characteristics of the Study Participants [1]

Injury Group [2]	No Surgery				Extracranial Surgery [3]				p-Value (Weighted Surgery Comparison) [4]			
	**m/sTBI** (GCS 3-12) (n=156)	**CT+TBI** (GCS 13-15) (n=546)	**CT-TBI** (GCS 13-15) (n=579)	**OTC** (n=68)	**m/sTBI** (GCS 3-12) (n=98)	**CT+TBI** (GCS 13-15) (n=86)	**CT-TBI** (GCS 13-15) (n=181)	**OTC** (n=121)	**m/sTBI**	**CT+TBI**	**CT-TBI**	**OTC**
**Time to ED**, mean (SD)	1.7 (3.0)	2.5 (3.2)	1.9 (3.1)	2.6 (3.7)	1.6 (1.7)	2.1 (2.8)	1.7 (2.9)	2.6 (3.1)	.069	.546	.749	.594
**ED Discharge**												
Ward	10 (6%)	222 (41%)	466 (80%)	58 (85%)	2 (2%)	20 (23%)	110 (61%)	107 (88%)	.212	.023	.010	---
ICU	146 (94%)	324 (59%)	113 (20%)	10 (15%)	96 (98%)	66 (77%)	71 (39%)	14 (12%)				
**Initial CT scan positive**												
**Subarachnoid Hemorrhage** [5]												
No	45 (31%)	185 (34%)	577 (100%)	29 (100%)	35 (38%)	26 (31%)	181 (100%)	24 (100%)	.607	.560	---	---
Yes (no other intracranial findings)	9 (6%)	93 (17%)	2 (0%)	0 (0%)	7 (8%)	15 (18%)	0 (0%)	0 (0%)				
Yes (with only skull fracture)	1 (1%)	18 (3%)	0 (0%)	0 (0%)	4 (4%)	6 (7%)	0 (0%)	0 (0%)				
Yes (with other intracranial injuries)	90 (62%)	250 (46%)	0 (0%)	0 (0%)	46 (50%)	38 (45%)	0 (0%)	0 (0%)				
**Subdural Hematoma**												
No	73 (50%)	283 (52%)	579 (100%)	29 (100%)	49 (53%)	47 (56%)	181 (100%)	24 (100%)	.247	.153	---	---
Yes (no other intracranial findings)	1 (1%)	52 (10%)	0 (0%)	0 (0%)	5 (5%)	11 (13%)	0 (0%)	0 (0%)				
Yes (with only skull fracture)	1 (1%)	8 (1%)	0 (0%)	0 (0%)	0 (0%)	0 (0%)	0 (0%)	0 (0%)				
Yes (with other intracranial injuries)	70 (48%)	203 (37%)	0 (0%)	0 (0%)	38 (41%)	26 (31%)	0 (0%)	0 (0%)				
**Epidural Hematoma**												
No	133 (92%)	476 (87%)	579 (100%)	29 (100%)	86 (93%)	78 (93%)	181 (100%)	25 (100%)	.710	.658	---	---
Yes (no other intracranial findings)	0 (0%)	1 (0%)	0 (0%)	0 (0%)	0 (0%)	0 (0%)	0 (0%)	0 (0%)				
Yes (with only skull fracture)	0 (0%)	21 (4%)	0 (0%)	0 (0%)	0 (0%)	2 (2%)	0 (0%)	0 (0%)				
Yes (with other intracranial injuries)	12 (8%)	48 (9%)	0 (0%)	0 (0%)	6 (7%)	4 (5%)	0 (0%)	0 (0%)				
**Contusions**												
No	82 (57%)	359 (66%)	579 (100%)	29 (100%)	62 (67%)	64 (76%)	181 (100%)	24 (100%)	.576	.217	---	---
Yes (no other intracranial findings)	1 (1%)	14 (3%)	0 (0%)	0 (0%)	0 (0%)	3 (4%)	0 (0%)	0 (0%)				
Yes (with only skull fracture)	0 (0%)	7 (1%)	0 (0%)	0 (0%)	3 (3%)	1 (1%)	0 (0%)	0 (0%)				
Yes (with other intracranial injuries)	62 (43%)	166 (30%)	0 (0%)	0 (0%)	27 (29%)	16 (19%)	0 (0%)	0 (0%)				

© 2025 Roberts CJ et al. *JAMA Network Open*.

eTable 1. Clinical and Demographic Characteristics of the Study Participants [1]

Injury Group [2]	No Surgery				Extracranial Surgery [3]				p-Value (Weighted Surgery Comparison) [4]			
	**m/sTBI** (GCS 3-12) (n=156)	**CT+TBI** (GCS 13-15) (n=546)	**CT-TBI** (GCS 13-15) (n=579)	**OTC** (n=68)	**m/sTBI** (GCS 3-12) (n=98)	**CT+TBI** (GCS 13-15) (n=86)	**CT-TBI** (GCS 13-15) (n=181)	**OTC** (n=121)	**m/sTBI**	**CT+TBI**	**CT-TBI**	**OTC**
**Intraventricular Hemorrhage**												
No	122 (84%)	512 (94%)	579 (100%)	29 (100%)	74 (80%)	81 (95%)	181 (100%)	24 (100%)	.640	.552	---	---
Yes (no other intracranial findings)	2 (1%)	5 (1%)	0 (0%)	0 (0%)	1 (1%)	1 (1%)	0 (0%)	0 (0%)				
Yes (with only skull fracture)	0 (0%)	0 (0%)	0 (0%)	0 (0%)	0 (0%)	0 (0%)	0 (0%)	0 (0%)				
Yes (with other intracranial injuries)	21 (14%)	29 (5%)	0 (0%)	0 (0%)	17 (18%)	3 (4%)	0 (0%)	0 (0%)				
**Shear**												
No	109 (75%)	476 (87%)	579 (100%)	29 (100%)	58 (63%)	72 (86%)	181 (100%)	24 (100%)	.236	.056	---	---
Yes (no other intracranial findings)	2 (1%)	33 (6%)	0 (0%)	0 (0%)	4 (4%)	5 (6%)	0 (0%)	0 (0%)				
Yes (with only skull fracture)	0 (0%)	0 (0%)	0 (0%)	0 (0%)	2 (2%)	0 (0%)	0 (0%)	0 (0%)				
Yes (with other intracranial injuries)	34 (23%)	37 (7%)	0 (0%)	0 (0%)	28 (30%)	7 (8%)	0 (0%)	0 (0%)				
**Skull Fracture** [5]												
No	76 (53%)	332 (63%)	540 (96%)	28 (100%)	53 (58%)	57 (70%)	163 (96%)	24 (100%)	.689	.146	.784	---
Yes (no other intracranial findings)	2 (1%)	5 (1%)	21 (4%)	0 (0%)	1 (1%)	1 (1%)	7 (4%)	0 (0%)				
Yes (with other intracranial injuries)	65 (45%)	191 (36%)	0 (0%)	0 (0%)	37 (41%)	23 (28%)	0 (0%)	0 (0%)				
**Number of Surgeries**												
Mean (SD)					2.31 (2.24)	1.81 (1.30)	1.53 (1.36)	1.38 (0.90)	---	---	---	---
1					48 (49%)	48 (56%)	138 (76%)	92 (76%)				
2+					50 (51%)	38 (44%)	43 (24%)	29 (24%)				
**Time to 1st Surgery**												
Mean (SD) days					3.7 (4.5)	3.0 (4.4)	1.6 (1.9)	1.1 (1.3)	---	---	---	---
0 to <24 hrs					37 (38%)	28 (33%)	90 (50%)	86 (71%)				
24 to <72 hrs					22 (22%)	33 (38%)	67 (37%)	27 (22%)				
72 to <120 hrs					16 (16%)	13 (15%)	13 (7%)	6 (5%)				
120+ hrs					23 (23%)	12 (14%)	10 (6%)	2 (2%)				

© 2025 Roberts CJ et al. *JAMA Network Open*.

eTable 1. Clinical and Demographic Characteristics of the Study Participants [1]

Injury Group [2]	No Surgery				Extracranial Surgery [3]				p-Value (Weighted Surgery Comparison) [4]			
	**m/sTBI** (GCS 3-12) (n=156)	**CT+TBI** (GCS 13-15) (n=546)	**CT-TBI** (GCS 13-15) (n=579)	**OTC** (n=68)	**m/sTBI** (GCS 3-12) (n=98)	**CT+TBI** (GCS 13-15) (n=86)	**CT-TBI** (GCS 13-15) (n=181)	**OTC** (n=121)	**m/sTBI**	**CT+TBI**	**CT-TBI**	**OTC**
**Total Anesthesia Time** [6]												
Mean (SD) hours					4.2 (4.0)	3.9 (3.6)	3.3 (3.2)	2.6 (2.3)	---	---	---	---
0 to <2 hrs					26 (30%)	26 (36%)	76 (44%)	60 (52%)				
2 to <6 hrs					45 (51%)	36 (49%)	79 (46%)	46 (40%)				
6+ hrs					17 (19%)	11 (15%)	17 (10%)	10 (9%)				
**Total ICU LOS**, mean (SD), d	5.9 (8.2)	1.2 (2.1)	0.4 (1.2)	0.3 (1.1)	10.8 (9.4)	5.7 (8.2)	1.4 (4.1)	0.5 (1.9)	<.001	<.001	.020	.459
**Discharged Home**												
Yes	73 (48%)	465 (87%)	541 (95%)	60 (92%)	31 (33%)	45 (54%)	148 (83%)	107 (90%)	.229	<.001	<.001	.607
**Withdrawal of Care**												
Yes	18 (12%)	7 (1%)	0 (0%)	0 (0%)	4 (4%)	1 (1%)	0 (0%)	0 (0%)	.041	.602	---	---
**Surgery Type**												
Maxillofacial					19 (19%)	11 (13%)	30 (17%)	1 (1%)	---	---	---	---
Extremity fracture lower limb (internal fixation)					22 (22%)	17 (20%)	44 (24%)	43 (36%)	---	---	---	---
Extremity fracture lower limb (external fixation)					8 (8%)	4 (5%)	16 (9%)	12 (10%)	---	---	---	---
Extremity fracture upper limb (internal fixation)					18 (18%)	17 (20%)	37 (20%)	23 (19%)	---	---	---	---
Extremity fracture upper limb (external fixation)					0 (0%)	2 (2%)	3 (2%)	5 (4%)	---	---	---	---
Fasciotomy					0 (0%)	0 (0%)	3 (2%)	0 (0%)	---	---	---	---
Laparotomy (abdomen)					17 (17%)	5 (6%)	7 (4%)	0 (0%)	---	---	---	---
Pelvic fracture (internal fixation)					7 (7%)	8 (9%)	11 (6%)	4 (3%)	---	---	---	---
Pelvic fracture (external fixation)					3 (3%)	2 (2%)	1 (1%)	0 (0%)	---	---	---	---
Spinal stabilisation/cervical					1 (1%)	8 (9%)	9 (5%)	0 (0%)	---	---	---	---
Spinal stabilisation/thoracic					3 (3%)	1 (1%)	3 (2%)	0 (0%)	---	---	---	---
Spinal stabilisation/lumbar					2 (2%)	1 (1%)	3 (2%)	0 (0%)	---	---	---	---
Thoracotomy					2 (2%)	0 (0%)	1 (1%)	2 (2%)	---	---	---	---

© 2025 Roberts CJ et al. *JAMA Network Open*.

**eTable 1.** Clinical and Demographic Characteristics of the Study Participants [1]												
**Injury Group** [2]	**No Surgery**				**Extracranial Surgery** [3]				**p-Value** (Weighted Surgery Comparison) [4]			
	**m/sTBI** (GCS 3-12) (n=156)	**CT+TBI** (GCS 13-15) (n=546)	**CT-TBI** (GCS 13-15) (n=579)	**OTC** (n=68)	**m/sTBI** (GCS 3-12) (n=98)	**CT+TBI** (GCS 13-15) (n=86)	**CT-TBI** (GCS 13-15) (n=181)	**OTC** (n=121)	**m/sTBI**	**CT+TBI**	**CT-TBI**	**OTC**
Tracheostomy					20 (20%)	6 (7%)	1 (1%)	0 (0%)	---	---	---	---
Vascular (operative)					1 (1%)	5 (6%)	1 (1%)	1 (1%)	---	---	---	---
Vascular (endovascular treatment)					4 (4%)	1 (1%)	1 (1%)	1 (1%)	---	---	---	---
Wound closure/graft					9 (9%)	7 (8%)	15 (8%)	20 (17%)	---	---	---	---
Other Extracranial					36 (37%)	33 (38%)	46 (25%)	31 (26%)	---	---	---	---

Comparisons quantified between No Surgery and Extracranial Surgery groups. Sub-groups included moderate/severe TBI (m/sTBI), CT positive TBI (+CT scan; CT+TBI), CT negative TBI (-CT scan; CT-TBI), or participants with peripheral orthopedic traumatic injuries (orthopedic trauma control [OTC] group). Abbreviations: standard deviation (SD), emergency department (ED), intensive care unit (ICU), and length of stay (LOS).

[1] Data are number (percentage) of participants unless otherwise specified.

[2] Surgical Comparison p-value is for 'No Surgery' versus 'Extracranial Surgery' contrasts within groups based on injury severity.

[3] Extracranial surgery refers to all surgeries outside of the skull, including maxillofacial procedures. All participants that underwent intracranial procedures (i.e. craniotomy) were excluded.

[4] Weighted by inverse probability of injury group membership.

[5] There were 2 participants whose CT scans were initially unavailable or read as negative but were subsequently updated to identify a subarachnoid hemorrhage that were not reassigned groups. CT scans with isolated skull fracture and no other findings were classified as negative, with 21 and 7 participants in the No Surgery and EC Surgery groups meeting that criteria and therefore assigned to the CT-TBI (GCS 13-15) injury subgroup.

[6] The calculation of total surgery time collapses concurrent surgeries when the start and stop times are identical. Subjects with any data-entry anomalies in their surgery record, including surgery times that partially overlap, are considered to have unknown surgery time.

© 2025 Roberts CJ et al. *JAMA Network Open*.

eTable 2. Clinical and Demographic Characteristics of the Study Participants

	**No Surgery** n = 1349	**Extracranial Surgery[1]** n = 486	**p-Value[2]** (Surgery Comparison)	
			**Unwt.**	**Wt.[3]**
**Injury Group** [4]				
m/sTBI	156 (12%)	98 (20%)	<.001	<.001
CT+TBI	546 (40%)	86 (18%)		
CT-TBI	579 (43%)	181 (37%)		
OTC	68 (5%)	121 (25%)		
GCS score, mean (SD) [5]	13.7 (3.0)	12.2 (4.4)	.028	.148
**Age**, mean (SD), y	42.7 (18.3)	40.6 (16.3)	.085	.115
**Sex**				
Male	914 (68%)	365 (75%)	.003	.002
Female	435 (32%)	121 (25%)		
**Race**				
White	1034 (78%)	378 (80%)	.333	.675
Black	222 (17%)	77 (16%)		
Other	77 (6%)	19 (4%)		
**Hispanic**	273 (21%)	120 (25%)	.039	.273
**Education**, mean (SD), y	13.3 (3.0)	13.0 (2.8)	.023	.448
**Injury Cause**			<.001	.014
MVC Occupant	409 (30%)	159 (33%)		
MCC	115 (9%)	78 (16%)		
MVC (cyclist or pedestrian)	178 (13%)	64 (13%)		
Fall	395 (29%)	97 (20%)		
Assault	95 (7%)	11 (2%)		
Other or Unknown	157 (12%)	77 (16%)		
**Loss of Consciousness**	1087 (85%)	316 (67%)	<.001	<.001
**Posttraumatic Amnesia**	959 (78%)	250 (60%)	<.001	<.001
**Initial CT scan positive** [6]	665 (51%)	168 (44%)	.011	.028
**Subarachnoid Hemorrhage**			.062	.175
No	836 (64%)	266 (70%)		
Yes (no other intracranial findings)	104 (8%)	22 (6%)		
Yes (with only skull fracture)	19 (1%)	10 (3%)		
Yes (with other intracranial injuries)	340 (26%)	84 (22%)		
**Subdural Hematoma**			.059	.221
No	964 (74%)	301 (79%)		
Yes (no other intracranial findings)	53 (4%)	16 (4%)		
Yes (with only skull fracture)	9 (1%)	0 (0%)		
Yes (with other intracranial injuries)	273 (21%)	64 (17%)		

© 2025 Roberts CJ et al. *JAMA Network Open*.

eTable 2. Clinical and Demographic Characteristics of the Study Participants

	No Surgery n = 1349	Extracranial Surgery[1] n = 486	p-Value[2] (Surgery Comparison)	
			Unwt.	Wt.[3]
**Epidural Hematoma**			.019	.299
No	1217 (94%)	370 (97%)		
Yes (no other intracranial findings)	1 (0%)	0 (0%)		
Yes (with only skull fracture)	21 (2%)	2 (1%)		
Yes (with other intracranial injuries)	60 (5%)	10 (3%)		
**Contusions**			.005	.119
No	1049 (81%)	331 (87%)		
Yes (no other intracranial findings)	15 (1%)	3 (1%)		
Yes (with only skull fracture)	7 (1%)	4 (1%)		
Yes (with other intracranial injuries)	228 (18%)	43 (11%)		
**Intraventricular Hemorrhage**			.263	.910
No	1242 (96%)	360 (94%)		
Yes (no other intracranial findings)	7 (1%)	2 (1%)		
Yes (with only skull fracture)	0 (0%)	0 (0%)		
Yes (with other intracranial injuries)	50 (4%)	20 (5%)		
**Shear**			.016	.006
No	1193 (92%)	335 (88%)		
Yes (no other intracranial findings)	35 (3%)	9 (2%)		
Yes (with only skull fracture)	0 (0%)	2 (1%)		
Yes (with other intracranial injuries)	71 (5%)	35 (9%)		
**Skull Fracture** [7]			.120	.321
No	976 (77%)	297 (81%)		
Yes (no other intracranial findings)	28 (2%)	9 (2%)		
Yes (with other intracranial injuries)	256 (20%)	60 (16%)		
**Time to ED**, mean (SD), h	2.1 (3.2)	2.0 (2.7)	.404	.382
**ED Discharge** [8]			.009	.596
Ward	756 (56%)	239 (49%)		
ICU	593 (44%)	247 (51%)		
**ISS Total**				
Mean (SD)	11.9 (8.2)	15.9 (11.3)	<.001	.715
**ISS Peripheral**				
Mean (SD)	4.6 (5.9)	10.3 (8.3)	<.001	<.001
**Max AIS Peripheral**			<.001	<.001
Mean (SD)	1.5 (1.1)	2.4 (1.0)		
0	242 (19%)	13 (3%)		
1	489 (38%)	57 (12%)		
2	300 (23%)	183 (38%)		

© 2025 Roberts CJ et al. *JAMA Network Open*.

**eTable 2.** Clinical and Demographic Characteristics of the Study Participants				
	**No Surgery n = 1349**	**Extracranial Surgery[1] n = 486**	**p-Value[2]** (Surgery Comparison)	
			**Unwt.**	**Wt.[3]**
3	210 (16%)	177 (37%)		
4	36 (3%)	36 (8%)		
5	2 (0%)	10 (2%)		
**Shock Index** [9]				
Mean (SD)	0.65 (0.19)	0.69 (0.22)	.002	.161
**Hemoglobin** [9]				
Mean (SD), g/dL	14.1 (1.6)	13.8 (1.7)	.045	.230
**SpO2** [9]				
Mean (SD), %	97.9 (2.7)	97.7 (3.3)	.750	.505
**Base Deficit** [9]				
Mean (SD), mEq/L	2.7 (3.2)	3.8 (3.7)	.004	.062
**Number of Surgeries**				
Mean (SD)		1.70 (1.52)	---	---
1		326 (67%)		
2+		160 (33%)		
**Time to 1st Surgery**				
Mean (SD) days		2.1 (3.2)	---	---
0 to <24 hrs		241 (50%)		
24 to <72 hrs		149 (31%)		
72 to <120 hrs		48 (10%)		
120+ hrs		47 (10%)		
**Total Anesthesia Time**				
Mean (SD) hours		3.4 (3.3)	---	---
0 to <2 hrs		188 (42%)		
2 to <6 hrs		206 (46%)		
6+ hrs		55 (12%)		
**Total ICU LOS** [10]				
Mean (SD), d	1.3 (3.5)	3.7 (7.1)	<.001	.002
**Discharged Home** [11]				
Yes	1139 (86%)	331 (70%)	<.001	<.001
**Withdrawal of Care**				
Yes	25 (2%)	5 (1%)	.297	.222
**Surgery Type**				
Maxillofacial		61 (13%)	---	---
Extremity fracture lower limb (internal fixation)		126 (26%)	---	---
Extremity fracture lower limb (external fixation)		40 (8%)	---	---

© 2025 Roberts CJ et al. *JAMA Network Open*.

**eTable 2.** Clinical and Demographic Characteristics of the Study Participants				
	**No Surgery** n = 1349	**Extracranial Surgery[1]** n = 486	**p-Value[2]** (Surgery Comparison)	
			**Unwt.**	**Wt.[3]**
Extremity fracture upper limb (internal fixation)		95 (20%)	---	---
Extremity fracture upper limb (external fixation)		10 (2%)	---	---
Fasciotomy		3 (1%)	---	---
Laparotomy (abdomen)		29 (6%)	---	---
Pelvic fracture (internal fixation)		30 (6%)	---	---
Pelvic fracture (external fixation)		6 (1%)	---	---
Spinal stabilisation/cervical		18 (4%)	---	---
Spinal stabilisation/thoracic		7 (1%)	---	---
Spinal stabilisation/lumbar		6 (1%)	---	---
Thoracotomy		5 (1%)	---	---
Tracheostomy		27 (6%)	---	---
Vascular (operative)		8 (2%)	---	---
Vascular (endovascular treatment)		7 (1%)	---	---
Wound closure/graft		51 (10%)	---	---
Other Extracranial		146 (30%)	---	---

Comparisons quantified between No Surgery and Extracranial Surgery groups. Abbreviations: standard deviation (SD), motor vehicle crash (MVC), motorcycle crash (MCC), emergency department (ED), Injury Severity Score (ISS), Abbreviated Injury Scale (AIS), intensive care unit (ICU), and length of stay (LOS).

[1] Extracranial surgery refers to all surgeries outside of the skull, including maxillofacial procedures. All participants that underwent intracranial procedures (i.e. craniotomy) were excluded.

[2] Surgical Comparison p-value is for main effect of 'No Surgery' versus 'Extracranial Surgery' contrasts for group totals (not based on injury severity).

[3] Weighted by inverse probability of injury group membership.

[4] Data are number (percentage) of participants unless otherwise specified.

[5] GCS score based on TBI groups only, not including the OTC group.

[6] CT scan of head considered positive for acute intracranial findings not counting skull fractures in isolation (eResults).

[7] CT scans with isolated skull fracture and no other findings were classified as negative, with 21 and 7 participants in the No Surgery and EC Surgery groups meeting that criterion and therefore assigned to the CT-TBI (GCS 13-15) injury subgroup.

[8] ED discharge locations were either ICU or hospital ward since discharge to home was an exclusion criteria.

[9] First on admission within 24 hrs assessed with shock index, hemoglobin, oxygen saturation (SpO2), and base deficit.

[10] Total includes the sum of all ICU readmissions prior to discharge.

[11] Final discharge destination was either home or other locations, including nursing home, sub-acute rehabilitation facility, or participant was deceased.

© 2025 Roberts CJ et al. *JAMA Network Open*.

eTable 3. Standardized Cohen *d* Effect sizes from Regression Models for Functional, Neurocognitive, Disability, and Quality-of-Life Outcomes at 1 Year (Excluding Deaths Occurring Within 2 Weeks of Injury)[1]

1 Year Outcomes	GOSE-ALL		GOSE-TBI		Trails A		Trails B		DRS		QoLIBRI-OS	
	Cohen's d (95% CI)	p	Cohen's d (95% CI)	p	Cohen's d (95% CI)	p	Cohen's d (95% CI)	p	Cohen's d (95% CI)	p	Cohen's d (95% CI)	p
Injury group x Surgery	---	<.001	---	<.001	---	.555	---	.001	---	<.001	---	.032
EC Surgery (m/sTBI)	-0.94 (-1.25, -0.63)	<.001	-0.92 (-1.22, -0.62)	<.001	0.42 (-0.06, 0.91)	.087	1.01 (0.53, 1.49)	<.001	0.97 (0.60, 1.34)	<.001	-0.59 (-0.97, -0.20)	.003
EC Surgery (CT+TBI)	-0.52 (-0.79, -0.25)	<.001	-0.42 (-0.69, -0.16)	.002	0.18 (-0.18, 0.53)	.324	0.53 (0.17, 0.88)	.003	0.68 (0.36, 1.00)	<.001	0.03 (-0.30, 0.36)	.850
EC Surgery (CT-TBI)	-0.01 (-0.21, 0.18)	.897	0.01 (-0.18, 0.20)	.905	0.12 (-0.13, 0.37)	.334	0.07 (-0.17, 0.32)	.559	0.05 (-0.17, 0.27)	.660	0.04 (-0.17, 0.25)	.711
EC Surgery (OTC)	0.03 (-0.32, 0.37)	.878	N/A	N/A	-0.07 (-0.55, 0.41)	.779	-0.12 (-0.59, 0.36)	.634	0.02 (-0.39, 0.44)	.907	0.09 (-0.31, 0.48)	.674

[1] Standardized Cohen's d effect-size, calculated as the reported B estimate (in Table 2) divided by the standard deviation of the residuals, where values of 0.2, 0.5, and 0.8 are considered small, medium, and large effects, respectively, for exposure to extracranial surgery without intracranial surgery compared to non-surgical participants.

© 2025 Roberts CJ et al. *JAMA Network Open*.

eTable 4. Functional outcome assessments among TBI and OTC participants: Glasgow Outcome Scale-Extended (GOSE)

	No Surgery					Extracranial Surgery				
	Total	m/sTBI (GCS 3-12)	CT+TBI (GCS 13-15)	CT-TBI (GCS 13-15)	OTC	Total	m/sTBI (GCS 3-12)	CT+TBI (GCS 13-15)	CT-TBI (GCS 13-15)	OTC
**Subjects**	**1349**	**156**	**546**	**579**	**68**	**486**	**98**	**86**	**181**	**121**
**1yr GOSE-ALL**	N=841	N=100	N=358	N=343	N=40	N=308	N=69	N=56	N=106	N=77
Mean (SD)	6.6 (1.7)	4.9 (2.8)	6.7 (1.6)	6.9 (1.3)	7.2 (1.0)	6.3 (1.9)	4.7 (2.1)	6.0 (2.2)	6.9 (1.3)	7.2 (1.1)
1	47 (6%)	29 (29%)	14 (4%)	4 (1%)	0 (0%)	15 (5%)	9 (13%)	5 (9%)	1 (1%)	0 (0%)
2	0 (0%)	0 (0%)	0 (0%)	0 (0%)	0 (0%)	1 (0%)	0 (0%)	1 (2%)	0 (0%)	0 (0%)
3	6 (1%)	3 (3%)	1 (0%)	2 (1%)	0 (0%)	18 (6%)	14 (20%)	4 (7%)	0 (0%)	0 (0%)
4	12 (1%)	5 (5%)	6 (2%)	1 (0%)	0 (0%)	8 (3%)	6 (9%)	1 (2%)	1 (1%)	0 (0%)
5	88 (10%)	11 (11%)	34 (9%)	41 (12%)	2 (5%)	40 (13%)	12 (17%)	4 (7%)	16 (15%)	8 (10%)
6	151 (18%)	11 (11%)	66 (18%)	64 (19%)	10 (25%)	55 (18%)	16 (23%)	11 (20%)	16 (15%)	12 (16%)
7	225 (27%)	15 (15%)	110 (31%)	95 (28%)	5 (13%)	60 (19%)	6 (9%)	15 (27%)	26 (25%)	13 (17%)
8	312 (37%)	26 (26%)	127 (35%)	136 (40%)	23 (58%)	111 (36%)	6 (9%)	15 (27%)	46 (43%)	44 (57%)
**1yr GOSE-TBI**	N=801	N=100	N=358	N=343	N=0	N=231	N=69	N=56	N=106	N=0
Mean (SD)	6.6 (1.8)	5.0 (2.8)	6.8 (1.5)	7.0 (1.2)		6.2 (2.0)	4.8 (2.1)	6.2 (2.2)	7.2 (1.2)	
1	47 (6%)	29 (29%)	14 (4%)	4 (1%)		15 (6%)	9 (13%)	5 (9%)	1 (1%)	
2	0 (0%)	0 (0%)	0 (0%)	0 (0%)		1 (0%)	0 (0%)	1 (2%)	0 (0%)	
3	5 (1%)	3 (3%)	1 (0%)	1 (0%)		14 (6%)	11 (16%)	3 (5%)	0 (0%)	
4	10 (1%)	5 (5%)	4 (1%)	1 (0%)		9 (4%)	7 (10%)	1 (2%)	1 (1%)	
5	70 (9%)	9 (9%)	29 (8%)	32 (9%)		20 (9%)	10 (14%)	2 (4%)	8 (8%)	
6	134 (17%)	12 (12%)	63 (18%)	59 (17%)		41 (18%)	17 (25%)	9 (16%)	15 (14%)	
7	219 (27%)	16 (16%)	112 (31%)	91 (27%)		50 (22%)	8 (12%)	17 (30%)	25 (24%)	
8	316 (39%)	26 (26%)	135 (38%)	155 (45%)		81 (35%)	7 (10%)	18 (32%)	56 (53%)	
**1yr GOSE-ALL** Excluding Deaths within 2wk	N=806	N=74	N=350	N=342	N=40	N=300	N=65	N=53	N=105	N=77
Mean (SD)	6.8 (1.3)	6.3 (1.8)	6.8 (1.3)	6.9 (1.2)	7.2 (1.0)	6.4 (1.7)	4.9 (1.9)	6.2 (1.9)	7.0 (1.1)	7.2 (1.1)
1	12 (1%)	3 (4%)	6 (2%)	3 (1%)	0 (0%)	7 (2%)	5 (8%)	2 (4%)	0 (0%)	0 (0%)
2	0 (0%)	0 (0%)	0 (0%)	0 (0%)	0 (0%)	1 (0%)	0 (0%)	1 (2%)	0 (0%)	0 (0%)
3	6 (1%)	3 (4%)	1 (0%)	2 (1%)	0 (0%)	18 (6%)	14 (22%)	4 (8%)	0 (0%)	0 (0%)
4	12 (1%)	5 (7%)	6 (2%)	1 (0%)	0 (0%)	8 (3%)	6 (9%)	1 (2%)	1 (1%)	0 (0%)
5	88 (11%)	11 (15%)	34 (10%)	41 (12%)	2 (5%)	40 (13%)	12 (18%)	4 (8%)	16 (15%)	8 (10%)
6	151 (19%)	11 (15%)	66 (19%)	64 (19%)	10 (25%)	55 (18%)	16 (25%)	11 (21%)	16 (15%)	12 (16%)
7	225 (28%)	15 (20%)	110 (31%)	95 (28%)	5 (13%)	60 (20%)	6 (9%)	15 (28%)	26 (25%)	13 (17%)

© 2025 Roberts CJ et al. *JAMA Network Open*.

eTable 4. Functional outcome assessments among TBI and OTC participants: Glasgow Outcome Scale-Extended (GOSE)										
	No Surgery					Extracranial Surgery				
	Total	m/sTBI (GCS 3-12)	CT+TBI (GCS 13-15)	CT-TBI (GCS 13-15)	OTC	Total	m/sTBI (GCS 3-12)	CT+TBI (GCS 13-15)	CT-TBI (GCS 13-15)	OTC
8	312 (39%)	26 (35%)	127 (36%)	136 (40%)	23 (58%)	111 (37%)	6 (9%)	15 (28%)	46 (44%)	44 (57%)
**1yr GOSE-TBI** Excluding Deaths within 2wk	N=766	N=74	N=350	N=342	N=0	N=223	N=65	N=53	N=105	N=0
Mean (SD)	6.9 (1.3)	6.3 (1.8)	6.9 (1.3)	7.0 (1.2)		6.4 (1.8)	5.1 (1.9)	6.5 (1.8)	7.2 (1.0)	
1	12 (2%)	3 (4%)	6 (2%)	3 (1%)		7 (3%)	5 (8%)	2 (4%)	0 (0%)	
2	0 (0%)	0 (0%)	0 (0%)	0 (0%)		1 (0%)	0 (0%)	1 (2%)	0 (0%)	
3	5 (1%)	3 (4%)	1 (0%)	1 (0%)		14 (6%)	11 (17%)	3 (6%)	0 (0%)	
4	10 (1%)	5 (7%)	4 (1%)	1 (0%)		9 (4%)	7 (11%)	1 (2%)	1 (1%)	
5	70 (9%)	9 (12%)	29 (8%)	32 (9%)		20 (9%)	10 (15%)	2 (4%)	8 (8%)	
6	134 (17%)	12 (16%)	63 (18%)	59 (17%)		41 (18%)	17 (26%)	9 (17%)	15 (14%)	
7	219 (29%)	16 (22%)	112 (32%)	91 (27%)		50 (22%)	8 (12%)	17 (32%)	25 (24%)	
8	316 (41%)	26 (35%)	135 (39%)	155 (45%)		81 (36%)	7 (11%)	18 (34%)	56 (53%)	

Group means (SD) for GOSE-ALL and GOSE-TBI at 1-year post-injury based on injury group and exposure to extracranial surgery. Differences between total sample sizes in "Subjects" row and all other sample sizes in functional assessment rows represent unavailable data that was treated as missing values. Orthopedic trauma control (OTC) groups did not have GOSE-TBI assessments since there was no component of brain injury on functional limitations.

© 2025 Roberts CJ et al. *JAMA Network Open*.

eTable 5. Regression Models for Functional Outcomes at 1 Year, Including Deaths Occurring Within 2 Weeks of Injury[1]				
Outcome	1yr GOSE-ALL		1yr GOSE-TBI	
	B (95% CI)<br>Cohen's d (95% CI)	p	B (95% CI)<br>Cohen's d (95% CI)	p
Injury group[2]	---	<.001	---	<.001
m/sTBI (vs. CT-TBI)	-1.29 (-1.64, -0.94)	<.001	-1.40 (-1.76, -1.04)	<.001
CT+TBI (vs. CT-TBI)	-0.04 (-0.27, 0.19)	.742	-0.09 (-0.32, 0.15)	.458
OTC (vs. CT-TBI)	0.39 (-0.10, 0.87)	.117	N/A	
Injury group x Surgery [3]	---	.009	---	.014
EC Surgery (m/sTBI)	-0.78 (-1.26, -0.31)<br>-0.54 (-0.87, -0.21)	.001	-0.73 (-1.21, -0.24)<br>-0.49 (-0.81, -0.16)	.004
EC Surgery (CT+TBI)	-0.67 (-1.09, -0.24)<br>-0.46 (-0.75, -0.17)	.002	-0.57 (-1.00, -0.13)<br>-0.38 (-0.67, -0.09)	.011
EC Surgery (CT-TBI)	0.02 (-0.30, 0.34)<br>0.02 (-0.21, 0.24)	.892	0.05 (-0.28, 0.38)<br>0.03 (-0.19, 0.25)	.775
EC Surgery (OTC)	-0.01 (-0.58, 0.56)<br>-0.01 (-0.40, 0.39)	.973	N/A	
Age (per +10y)	-0.17 (-0.22, -0.11)	<.001	-0.14 (-0.20, -0.09)	<.001
Sex (female vs male)	-0.17 (-0.36, 0.02)	.083	-0.15 (-0.35, 0.06)	.154
Race	---	.004	---	.022
Black (vs. White)	-0.37 (-0.62, -0.13)	.003	-0.28 (-0.55, -0.02)	.038
Other (vs. White)	0.20 (-0.18, 0.59)	.293	0.33 (-0.08, 0.74)	.114
Injury Cause	---	.505	---	.660
Fall (vs. MVC)	0.12 (-0.10, 0.34)	.285	0.07 (-0.16, 0.31)	.542
Other/Unk. (vs. MVC)	-0.01 (-0.26, 0.23)	.918	-0.06 (-0.33, 0.21)	.646
Education Yrs (per +4y) [4]	0.24 (0.11, 0.37)	<.001	0.18 (0.04, 0.32)	.010
ISS Peripheral (per +1pts)	-0.01 (-0.02, 0.01)	.235	0.00 (-0.01, 0.02)	.682

[1] A fixed-effects linear regression model evaluated the association between injury group, surgery group, and injury x surgery interaction within each clinical outcome and is propensity-weighted for missing outcome and extracranial (EC) surgery group imbalance (age, sex, race, years of education, cause of injury, GCS, non-head or neck ISS score, total ISS score, ICU admission, and time from injury to admission). Standardized Cohen's d effect-size, calculated as the reported B estimate divided by the standard deviation of the residuals, where values of 0.2, 0.5, and 0.8 are considered small, medium, and large effects, respectively. 95% confidence intervals for these effect sizes are reported.
[2] The main effects of injury group presented reflect the effect in those without EC surgery.
[3] Main effect of EC Surgery entered in the model and is reflected in the Injury Group x EC-Surgery (CT-TBI) contrasts.

© 2025 Roberts CJ et al. *JAMA Network Open*.

[4] Years of education is a complicated variable used as a proxy to account for multifactorial issues related to socioeconomic status and cognitive potential, which is partially confounded in this study, including participants 17 years and older, since some subjects have not yet completed their years of education.

© 2025 Roberts CJ et al. *JAMA Network Open*.

eTable 6. Neurocognitive, Disability, and Quality-of-Life outcome assessments among TBI and OTC participants at 2 weeks, 6 months, and 1 year after TBI. [1]

	No Surgery					Extracranial Surgery				
	Total	m/sTBI (GCS 3-12)	CT+TBI (GCS 13-15)	CT-TBI (GCS 13-15)	OTC	Total	m/sTBI (GCS 3-12)	CT+TBI (GCS 13-15)	CT-TBI (GCS 13-15)	OTC
**Subjects** [2]	**1349**	**156**	**546**	**579**	**68**	**486**	**98**	**86**	**181**	**121**
**2wk DRS**	N=985	N=101	N=401	N=436	N=47	N=375	N=83	N=64	N=140	N=88
Mean (SD)	4.94 (6.22)	12.7 (10.9)	4.08 (5.33)	4.08 (4.20)	3.51 (2.98)	9.79 (8.99)	20.0 (10.0)	11.31 (8.94)	6.39 (4.77)	4.51 (3.34)
**2wk QoLIBRI**	N=987	N=62	N=409	N=467	N=49	N=325	N=31	N=52	N=146	N=96
Mean (SD)	54.4 (24.4)	62.8 (19.2)	55.6 (24.4)	52.0 (24.9)	55.8 (22.5)	50.7 (22.9)	48.5 (18.2)	45.3 (22.1)	49.6 (25.1)	55.9 (20.2)
**6mo DRS**	N=855	N=75	N=355	N=385	N=40	N=320	N=66	N=52	N=120	N=82
Mean (SD)	1.93 (3.32)	3.19 (5.38)	1.76 (2.87)	1.94 (3.23)	1.08 (2.20)	3.44 (5.75)	8.12 (8.76)	3.88 (6.23)	2.19 (3.21)	1.23 (2.01)
**6mo QoLIBRI**	N=878	N=69	N=363	N=407	N=39	N=310	N=50	N=52	N=123	N=85
Mean (SD)	64.3 (26.0)	70.8 (23.5)	65.3 (24.8)	62.0 (27.1)	68.1 (26.8)	67.0 (23.4)	61.3 (22.7)	65.9 (21.5)	67.8 (25.7)	69.9 (21.2)
**1yr Trails A**	N=605	N=39	N=263	N=276	N=27	N=213	N=34	N=35	N=87	N=57
Mean (SD)	27.5 (14.7)	26.9 (14.7)	29.9 (17.5)	25.5 (11.6)	25.3 (9.5)	28.7 (16.0)	36.1 (19.7)	31.8 (19.6)	27.6 (14.9)	24.2 (10.0)
**1yr Trails B**	N=603	N=39	N=263	N=274	N=27	N=213	N=34	N=35	N=87	N=57
Mean (SD)	72.6 (49.2)	70.4 (52.4)	80.4 (58.4)	66.4 (38.6)	63.9 (31.1)	77.7 (57.7)	108.7 (77.1)	91.2 (76.6)	71.0 (47.8)	61.2 (31.1)
**1yr DRS**	N=810	N=71	N=343	N=357	N=39	N=296	N=58	N=51	N=113	N=74
Mean (SD)	1.91 (3.43)	3.63 (5.94)	1.88 (3.14)	1.69 (3.03)	0.97 (1.78)	2.85 (5.04)	7.31 (7.56)	2.92 (5.38)	1.86 (3.07)	0.81 (1.48)
**1yr QoLIBRI**	N=821	N=65	N=349	N=367	N=40	N=291	N=48	N=48	N=117	N=78
Mean (SD)	66.7 (25.5)	74.0 (20.4)	67.0 (24.9)	64.9 (26.8)	68.5 (23.9)	67.9 (24.3)	57.5 (24.2)	71.1 (22.3)	68.4 (25.4)	71.5 (22.2)

[1] Group means (SD) for Trail Making Test Part A and B (Trails A and B; number of seconds to complete; lower score is faster/better), Disability Rating Scale (DRS; (lower score is better; range 0–29)) and Quality of Life after Brain Injury-Overall Scale (QoLIBRI-OS; higher score is better; range 0–100) at 2-weeks, 6-months, and 1-year after TBI based on injury group and exposure to extracranial surgery.

[2] Differences between total sample sizes in "Subjects" row and all other sample sizes in assessment rows represent unavailable data that was treated as missing values. The percentage of subjects deemed able to participate in testing was based on screening tests. The extracranial surgery groups generally had lower

© 2025 Roberts CJ et al. *JAMA Network Open*.

eTable 6. Neurocognitive, Disability, and Quality-of-Life outcome assessments among TBI and OTC participants at 2 weeks, 6 months, and 1 year after TBI. [1]

	No Surgery					Extracranial Surgery				
	Total	m/sTBI (GCS 3-12)	CT+TBI (GCS 13-15)	CT-TBI (GCS 13-15)	OTC	Total	m/sTBI (GCS 3-12)	CT+TBI (GCS 13-15)	CT-TBI (GCS 13-15)	OTC

percentages of subjects able to participate in the tests at 2-weeks suggesting that those groups had worse outcomes at that point in time because fewer were able to participate. Therefore, these comparisons tend to underestimate the difference in the effect size between extracranial surgery and no surgery groups.

© 2025 Roberts CJ et al. *JAMA Network Open*.

eTable 7. **Regression Models for Disability and Quality-of-Life Outcomes at 2 weeks and 6 months, Excluding Deaths Occurring Within 2 Weeks of Injury[1]**

Outcome	2wk DRS		2wk QoLIBRI-OS		6mo DRS		6mo QoLIBRI-OS	
	B (95% CI)<br>Cohen's d (95% CI)	p	B (95% CI)<br>Cohen's d (95% CI)	p	B (95% CI)<br>Cohen's d (95% CI)	p	B (95% CI)<br>Cohen's d (95% CI)	p
Injury group[2]	---	<.001	---	.332	---	<.001	---	.035
m/sTBI (vs. CT-TBI)	9.59 (8.26, 10.93)	<.001	7.94 (1.51, 14.38)	.016	2.16 (1.19, 3.14)	<.001	4.54 (-1.75, 10.83)	.157
CT+TBI (vs. CT-TBI)	-0.26 (-1.13, 0.61)	.561	1.86 (-1.48, 5.20)	.274	-0.18 (-0.78, 0.42)	.561	1.28 (-2.43, 4.98)	.501
OTC (vs. CT-TBI)	-1.53 (-3.39, 0.33)	.107	5.58 (-1.55, 12.70)	.125	-1.29 (-2.58, 0.01)	.051	10.31 (2.15, 18.46)	.013
Injury group x Surgery[3]	---	<.001	---	.123	---	<.001	---	.052
EC Surgery (m/sTBI)	4.33 (2.52, 6.15)<br>0.75 (0.44, 1.07)	<.001	-11.27 (-21.38, -1.17)<br>-0.49 (-0.93, -0.05)	.029	3.93 (2.60, 5.27)<br>1.04 (0.69, 1.39)	<.001	-10.50 (-19.34, -1.66)<br>-0.43 (-0.80, -0.07)	.020
EC Surgery (CT+TBI)	5.56 (3.95, 7.17)<br>0.97 (0.69, 1.25)	<.001	-6.57 (-13.56, 0.43)<br>-0.28 (-0.59, 0.02)	.066	2.55 (1.36, 3.74)<br>0.67 (0.36, 0.99)	<.001	0.06 (-7.67, 7.78)<br>0.00 (-0.32, 0.32)	.988
EC Surgery (CT-TBI)	1.14 (-0.02, 2.30)<br>0.20 (0.00, 0.40)	.053	0.26 (-4.15, 4.67)<br>0.01 (-0.18, 0.20)	.908	0.16 (-0.65, 0.97)<br>0.04 (-0.17, 0.26)	.699	3.43 (-1.57, 8.42)<br>0.14 (-0.06, 0.35)	.179
EC Surgery (OTC)	1.77 (-0.40, 3.95)<br>0.31 (-0.07, 0.69)	.111	-4.58 (-12.90, 3.74)<br>-0.20 (-0.56, 0.16)	.281	0.68 (-0.83, 2.19)<br>0.18 (-0.22, 0.58)	.377	-3.69 (-13.15, 5.77)<br>-0.15 (-0.54, 0.24)	.445
Age (per +10y)	0.68 (0.47, 0.88)	<.001	-0.45 (-1.26, 0.36)	.274	0.34 (0.20, 0.49)	<.001	-1.80 (-2.69, -0.90)	<.001
Sex (female vs male)	1.52 (0.79, 2.24)	<.001	-9.46 (-12.34, -6.58)	<.001	0.31 (-0.20, 0.81)	.232	-6.92 (-10.04, -3.79)	<.001
Race	---	.818	---	.003	---	.001	---	<.001
Black (vs. White)	-0.28 (-1.17, 0.62)	.545	-4.76 (-8.35, -1.17)	.009	1.20 (0.57, 1.83)	<.001	-9.14 (-13.07, -5.22)	<.001
Other (vs. White)	-0.18 (-1.64, 1.27)	.804	5.60 (-0.17, 11.36)	.057	-0.38 (-1.40, 0.64)	.467	3.56 (-2.83, 9.95)	.274
Injury Cause	---	.179	---	.008	---	.388	---	.204
Fall (vs. MVC)	-0.76	.072	5.18	.002	-0.32	.278	2.05	.265

© 2025 Roberts CJ et al. *JAMA Network Open*.

	(-1.58, 0.07)		(1.89, 8.47)		(-0.90, 0.26)		(-1.56, 5.66)	
Other/Unk. (vs. MVC)	-0.07 (-0.99, 0.85)	.879	2.75 (-0.90, 6.40)	.141	0.15 (-0.49, 0.78)	.651	-1.94 (-5.92, 2.04)	.340
Education Yrs (per +4y) [4]	-0.63 (-1.09, -0.17)	.008	1.62 (-0.20, 3.43)	.082	-0.59 (-0.91, -0.27)	<.001	5.43 (3.41, 7.45)	<.001
ISS Peripheral (per +1pts)	0.16 (0.11, 0.21)	<.001	-0.16 (-0.37, 0.05)	.133	0.02 (-0.02, 0.06)	.298	0.06 (-0.16, 0.29)	.597

[1] A fixed-effects linear regression model evaluated the association between injury group, surgery group, and injury x surgery interaction within each clinical outcome and is propensity-weighted for missing outcome and extracranial (EC) surgery group imbalance (age, sex, race, education years, injury cause, and non-head or neck ISS score). Each timepoint was accounted for in a separate model. Standardized Cohen's d effect-size, calculated as the reported B estimate divided by the standard deviation of the residuals, where values of 0.2, 0.5, and 0.8 are considered small, medium, and large effects, respectively. 95% confidence intervals for these effect sizes are reported.

[2] The main effects of injury group presented reflect the effect in those without EC surgery.

[3] Main effect of EC Surgery entered in the model and is reflected in the Injury Group x EC-Surgery (CT-TBI) contrasts.

[4] Years of education is a complicated variable used as a proxy to account for multifactorial issues related to socioeconomic status and cognitive potential, which is partially confounded in this study, including participants 17 years and older, since some subjects have not yet completed their years of education.

© 2025 Roberts CJ et al. *JAMA Network Open*.

eTable 8. Regression Models[1] for Functional, Neurocognitive, Disability, and Quality-of-Life Outcomes at 1 Year, Excluding Deaths Occurring Within 2 Weeks of Injury, with robust estimation of SEs.

1 Year Outcomes	GOSE-ALL		GOSE-TBI		Trails A		Trails B		DRS		QoLIBRI-OS	
	B (95% CI)	p	B (95% CI)	p	B (95% CI)	p	B (95% CI)	p	B (95% CI)	p	B (95% CI)	p
Injury group [2]	---	<.001	---	<.001	---	<.001	---	<.001	---	<.001	---	.065
m/sTBI (vs. CT-TBI)	-0.55 (-0.86, -0.24)	<.001	-0.62 (-0.92, -0.31)	<.001	3.04 (-1.29, 7.37)	.169	7.7 (-7.2, 22.7)	.312	2.64 (1.65, 3.63)	<.001	4.01 (-2.51, 10.53)	.228
CT+TBI (vs. CT-TBI)	-0.07 (-0.26, 0.11)	.454	-0.13 (-0.31, 0.06)	.173	3.68 (1.40, 5.96)	.002	12.0 (4.1, 19.9)	.003	0.02 (-0.57, 0.62)	.937	1.10 (-2.71, 4.90)	.573
OTC (vs. CT-TBI)	0.24 (-0.15, 0.63)	.231	N/A	N/A	-0.09 (-5.23, 5.05)	.972	2.9 (-14.8, 20.6)	.747	-0.83 (-2.10, 0.44)	.201	4.72 (-3.39, 12.83)	.254
Injury group x Surgery [3]	---	.002	---	.002	---	.321	---	.042	---	.068	---	.027
EC Surgery (m/sTBI)	-1.28 (-1.94, -0.62)	<.001	-1.25 (-1.92, -0.59)	<.001	6.64 (0.14, 13.13)	.045	47.9 (5.8, 90.0)	.026	3.53 (0.35, 6.71)	.029	-15.11 (-24.73, -5.49)	.002
EC Surgery (CT+TBI)	-0.69 (-1.31, -0.07)	.029	-0.57 (-1.19, 0.04)	.068	2.24 (-2.89, 7.36)	.392	22.7 (3.5, 42.0)	.021	2.47 (-0.08, 5.02)	.057	0.72 (-6.89, 8.33)	.853
EC Surgery (CT-TBI)	-0.02 (-0.33, 0.30)	.918	0.02 (-0.28, 0.31)	.918	1.47 (-1.19, 4.14)	.278	3.4 (-5.8, 12.7)	.467	0.19 (-0.51, 0.88)	.593	1.03 (-5.79, 7.84)	.767
EC Surgery (OTC)	0.02 (-0.41, 0.46)	.918	N/A	N/A	-1.03 (-5.81, 3.75)	.673	-6.0 (-24.3, 12.3)	.522	0.10 (-0.55, 0.76)	.753	1.75 (-6.92, 10.41)	.693
Age (per +10y)	-0.07 (-0.11, -0.02)	.003	-0.03 (-0.08, 0.01)	.165	3.64 (3.09, 4.19)	<.001	12.7 (10.8, 14.6)	<.001	0.31 (0.17, 0.46)	<.001	-1.58 (-2.50, -0.66)	.001
Sex (female vs male)	-0.23 (-0.38, -0.07)	.004	-0.21 (-0.37, -0.05)	.010	-0.46 (-2.42, 1.51)	.648	-5.0 (-11.8, 1.7)	.145	0.37 (-0.13, 0.87)	.144	-4.30 (-7.50, -1.09)	.009
Race	---	.001	---	.007	---	<.001	---	<.001	---	.056	---	.001
Black (vs. White)	-0.34 (-0.54, -0.14)	.001	-0.25 (-0.46, -0.04)	.022	7.97 (5.47, 10.46)	<.001	32.3 (23.6, 40.9)	<.001	0.60 (-0.05, 1.24)	.069	-7.61 (-11.72, -3.49)	<.001
Other/Unk (vs. White)	0.15 (-0.16, 0.45)	.336	0.31 (-0.01, 0.63)	.058	4.77 (1.09, 8.45)	.011	12.9 (0.2, 25.6)	.048	-0.67 (-1.65, 0.30)	.174	2.82 (-3.42, 9.05)	.376
Injury Cause	---	.253	---	.565	---	.206	---	.166	---	.206	---	.521
Fall (vs. MVC)	0.15 (-0.03, 0.33)	.098	0.10 (-0.09, 0.28)	.299	1.80 (-0.44, 4.03)	.115	7.1 (-0.6, 14.9)	.070	-0.51 (-1.08, 0.06)	.081	1.10 (-2.60, 4.79)	.561
Other/Unk. (vs. MVC)	0.06 (-0.14, 0.26)	.568	0.01 (-0.20, 0.22)	.944	1.68 (-0.87, 4.22)	.196	5.0 (-3.8, 13.8)	.263	-0.08 (-0.72, 0.57)	.818	-1.57 (-5.70, 2.55)	.455
Education Yrs (per +4y) [4]	0.27 (0.17, 0.37)	<.001	0.21 (0.10, 0.31)	<.001	-6.43 (-7.72, -5.14)	<.001	-25.8 (-30.2, -21.3)	<.001	-0.56 (-0.88, -0.23)	.001	4.57 (2.47, 6.67)	<.001
ISS Peripheral (per +1pts)	-0.01 (-0.02, 0.01)	.295	0.01 (-0.01, 0.02)	.330	0.16 (0.02, 0.30)	.030	0.30 (-0.2, 0.8)	.251	-0.03 (-0.07, 0.00)	.072	0.11 (-0.13, 0.34)	.377

[1] Regression models in this table use robust estimation of standard errors. This more conservative approach resulted in increased confidence intervals for nearly all comparisons. As such the CT+TBI participants that were exposed to EC Surgery compared to the No Surgery group was no longer significantly different for

© 2025 Roberts CJ et al. *JAMA Network Open*.

GOSE-TBI or DRS. All other comparisons that were statistically significant. These findings decrease the confidence that the effects in these groups is not influenced by unmodeled variance.

© 2025 Roberts CJ et al. *JAMA Network Open*.

**eTable 9. Participants Lost to Follow-up at 1 year after Trauma.**				
	**TBI**		**OTC**	
	**No Surgery**	**EC Surgery**	**No Surgery**	**EC Surgery**
**Subjects**	**1281**	**365**	**68**	**121**
GOSE-ALL	801 (63%)	231 (63%)	40 (59%)	77 (64%)
GOSE-TBI	801 (63%)	231 (63%)	---	---
Neuropsych	578 (45%)	157 (43%)	27 (40%)	57 (47%)
DRS	771 (60%)	222 (61%)	39 (57%)	74 (61%)
QoLIBRI-OS	781 (61%)	213 (58%)	40 (59%)	78 (64%)
At 1-year after the index admission, the OTC and TBI groups had similar rates of loss to follow-up.				

© 2025 Roberts CJ et al. *JAMA Network Open*.
