# Supplementary material for: One-Year Outcomes After Traumatic Brain Injury and Early Extracranial Surgery in the TRACK-TBI Study
Source: JAMA Netw Open. 2025 Oct 10;8(10):e2537271. doi: 10.1001/jamanetworkopen.2025.37271 (PMC12514633; doi:10.1001/jamanetworkopen.2025.37271)
Supplement: Supplement 2. — Data Sharing Statement [file jamanetwopen-e2537271-s002.pdf]

Data Sharing Statement

Roberts. One-Year Outcomes After Traumatic Brain Injury and Early Extracranial Surgery—A TRACK-TBI Study. *JAMA Netw Open*. Published October 10, 2025. doi:10.1001/jamanetworkopen.2025.37271

Data
Data available: Yes
Data types: Deidentified participant data
How to access data: A Research Collaboration Proposal Request Form can be submitted to Dr. Geoffrey Manley, Contact PI for TRACK-TBI, in care of Brian Fabian at brian.fabian@ucsf.edu to be considered for collaboration.
When available: With publication

Supporting Documents
Document types: None

Additional Information
Who can access the data: Researchers whose proposed use of the data is approved by Dr. Geoffrey Manley and the TRACK-TBI Investigators.
Types of analyses: As specified in an approved Proposal Request Form.
Mechanisms of data availability: with a signed Data Transfer Agreement.
Any additional restrictions: N/A
